# TGF‐β1 mediates pathologic changes of secondary lymphedema by promoting fibrosis and inflammation

**DOI:** 10.1002/ctm2.758

**Published:** 2022-06-02

**Authors:** Jung Eun Baik, Hyeung Ju Park, Raghu P. Kataru, Ira L. Savetsky, Catherine L. Ly, Jinyeon Shin, Elizabeth M. Encarnacion, Michele R. Cavali, Mark G. Klang, Elyn Riedel, Michelle Coriddi, Joseph H. Dayan, Babak J. Mehrara

**Affiliations:** ^1^ Plastic and Reconstructive Surgery Service, Department of Surgery Memorial Sloan Kettering Cancer Center New York New York

**Keywords:** fibrosis, inflammation, pirfenidone, TGF‐β

## Abstract

**Background:**

Secondary lymphedema is a common complication of cancer treatment, and previous studies have shown that the expression of transforming growth factor‐beta 1 (TGF‐β1), a pro‐fibrotic and anti‐lymphangiogenic growth factor, is increased in this disease. Inhibition of TGF‐β1 decreases the severity of the disease in mouse models; however, the mechanisms that regulate this improvement remain unknown.

**Methods:**

Expression of TGF‐β1 and extracellular matrix molecules (ECM) was assessed in biopsy specimens from patients with unilateral breast cancer‐related lymphedema (BCRL). The effects of TGF‐β1 inhibition using neutralizing antibodies or a topical formulation of pirfenidone (PFD) were analyzed in mouse models of lymphedema. We also assessed the direct effects of TGF‐β1 on lymphatic endothelial cells (LECs) using transgenic mice that expressed a dominant‐negative TGF‐β receptor selectively on LECs (LEC^DN‐RII^).

**Results:**

The expression of TGF‐β1 and ECM molecules is significantly increased in BCRL skin biopsies. Inhibition of TGF‐β1 in mouse models of lymphedema using neutralizing antibodies or with topical PFD decreased ECM deposition, increased the formation of collateral lymphatics, and inhibited infiltration of T cells. In vitro studies showed that TGF‐β1 in lymphedematous tissues increases fibroblast, lymphatic endothelial cell (LEC), and lymphatic smooth muscle cell stiffness. Knockdown of TGF‐β1 responsiveness in LEC^DN‐RII^ resulted in increased lymphangiogenesis and collateral lymphatic formation; however, ECM deposition and fibrosis persisted, and the severity of lymphedema was indistinguishable from controls.

**Conclusions:**

Our results show that TGF‐β1 is an essential regulator of ECM deposition in secondary lymphedema and that inhibition of this response is a promising means of treating lymphedema.

## Graphical abstract

- TGF‐β1 expression, fibroblast proliferation and ECM deposition are increased in lymphedematous skin.
- Tissue lysate of lymphedematous skin induces fibroblast proliferation, ECM production and increases the stiffness of fibroblasts, LECs and LSMCs.
- Inhibition of TGF‐β1 decreases ECM deposition, immune cell infiltration and increases collateral lymphatics formation.
- Topical treatment of pirfenidone is highly effective for lymphedema treatment.

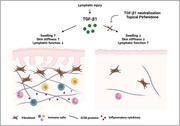

## 1 INTRODUCTION

Secondary lymphedema is a common complication of cancer treatment that results from iatrogenic injury to the lymphatic channels that drain the skin.^1^, ^2^ Breast cancer, due to its high prevalence, is the well‐known cause of lymphedema^3^; however, lymphedema also occurs commonly in other solid tumors such as sarcoma, melanoma and urologic and gynecologic cancers.^1^, ^4^, ^5^, ^6^, ^7^ Patients with lymphedema have lifelong and progressive skin fibroadipose tissue deposition that cause pain, chronic swelling and reduction of function.^8^, ^9^, ^10^, ^11^, ^12^ Many patients also develop recurrent skin infections requiring hospitalisation for intravenous antibiotics.^2^ Although lymphedema is common and morbid, current treatments are palliative, using physical therapy and compression garments to reduce symptoms and prevent progression of disease.^13^ Experimental treatments of lymphedema have centred around delivery of lymphangiogenic growth factor such as vascular endothelial growth factor‐C (VEGF‐C); however, these efforts have been largely abandoned due to equivocal outcomes in clinical trials.^14^, ^15^, ^16^, ^17^, ^18^ As a result, developing novel treatments for lymphedema is an important and significant goal.

Recent studies have shown that fibrosis is both a phenotype and a driving force in lymphedema pathogenesis. Analyses of clinical specimens have shown that lymphedema results in progressive collecting lymphatic vessel fibrosis, smooth muscle cell proliferation and lymphatic vessel luminal obstruction.^19^, ^20^, ^21^ There is also fibroadipose tissue deposition with increased deposition of type I and III collagen.^22^, ^23^ These findings are supported by magnetic resonance imaging in patients with lymphedema that show disorganisation and fibrosis of lymphatics and ECM deposition in late‐stage lymphedema.^24^ Capillary lymphatic vessels also become encased in fibrotic ECM and display a dilated, dysfunctional morphology.^13^ Excess fibrosis in various experimental settings, such as obesity, wound healing and radiation‐induced lymphatic injury, decreases lymphatic regeneration and function.^25^, ^26^, ^27^ In addition, inhibition of fibrosis, either by decreasing chronic inflammation or inhibition of pro‐fibrotic growth factors, improves lymphatic function and decreases the severity of lymphedema in mouse models.^13^, ^28^, ^29^

Transforming growth factor‐beta 1 (TGF‐β1) is an essential regulator of fibrosis that promotes ECM deposition by directly increasing fibroblast collagen production, decreasing turnover of matrix products and modulating inflammatory responses.^30^, ^31^ TGF‐β1 also directly inhibits lymphangiogenesis in a variety of settings.^26^, ^32^ The expression of TGF‐β1 is increased in lymphedematous tissues, and inhibition of this growth factor increases lymphangiogenesis and formation of collateral lymphatics and decreases swelling in mouse models of lymphedema.^13^, ^23^, ^26^, ^33^, ^34^ These findings suggest that, similar to other fibrotic disorders, the expression of TGF‐β1 in lymphedema inhibits lymphangiogenesis and promotes fibrosis and replacement of lymphatic vessels with scar tissue, eventually resulting in end‐organ failure of the lymphatic system.

However, while the literature strongly suggests that TGF‐β plays a role in the pathology of lymphedema by regulating fibrosis and inhibiting lymphangiogenesis, the changes in TGF‐β isoform expression and downstream pathways in clinical lymphedema samples have not been analysed. In addition, it is not clear if TGF‐β inhibition improves lymphedema by decreasing fibrosis, increasing lymphangiogenesis or both. In the present study, we analysed the expression of TGF‐β1 and downstream signaling pathways in patients with unilateral upper extremity breast cancer‐related lymphedema (BCRL) and show that these pathways are strongly activated in the diseased limb. Inhibition of TGF‐β1 using a monoclonal neutralising antibody improved lymphangiogenesis, decreased fibrosis and decreased inflammation in a mouse model. TGF‐β1 in lymphatic fluid increased proliferation and fibrotic protein expression by fibroblasts and increased cellular stiffness of fibroblasts, lymphatic endothelial cells (LECs) and lymphatic smooth muscle cells (LSMCs). Transgenic mice that expressed a dominant‐negative TGF‐β‐receptor II (TGF‐βRII) in LECs had significantly increased lymphangiogenesis and lymphatic collateral vessel formation but did not have improvements in tail lymphedema, suggesting that pathological effects of TGF‐β1 in lymphedema are mediated by changes in the ECM or modulation of inflammatory cell infiltration/function rather than anti‐lymphangiogenic effects. Finally, we show that a topical formulation of pirfenidone (PFD), a small‐molecule inhibitor approved by the US Food and Drug Administration (FDA) for the treatment of idiopathic pulmonary fibrosis, significantly decreased TGF‐β1 signaling, reduced fibrosis and decreased lymphedema formation in a mouse model.

## 2 RESULTS

### 2.1 BCRL results in increased TGF‐β1 expression and signaling

Using immunohistochemistry, previous papers have shown that the expression of TGF‐β1 was increased in tissue sections collected from a small number of patients with lymphedema.^13^, ^33^ To confirm these findings and study other TGF‐β isoforms, we collected matched upper extremity biopsies from 18 patients with unilateral BCRL (Table 1). These patients were all female and ranged in age from 48 to 68 years. Nearly 40% had a history of recurrent infections, and the duration of lymphedema ranged between 6 and 152 months. Most patients (77.8%) had a history of radiation either to the breast or to the regional lymph nodes.

**TABLE 1:** Patient demographics

	Value	Range
Patients (*n*)	18	–
Age (years)	56.6 ± 6.8	48–68
BMI (kg/m^2^)	25.3 ± 3.8	19.4–33.1
History of radiation therapy	77.8%	–
ISL Stage		
Stage I	5.3%	–
Stage II	94.7%	–
Duration of lymphedema (months)	68.8 ± 51.3	6–152
History of infections	38.9%	
Volume differential (cc)	791 ± 685	184–2519
Volume differential (%)	19.4 ± 9%	8.0–39.8
L‐Dex Score	25.4 ± 3.8	3.4–38.7

Abbreviations: BMI, body mass index; ISL, International Society of Lymphology; L‐Dex, Lymphedema Index.

**FIGURE 1:**
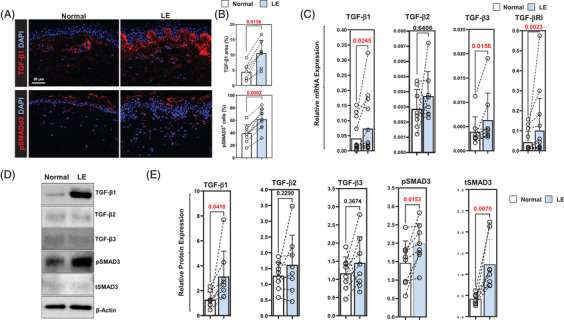
BCRL results in increased TGF‐β1 expression and signaling. (A) Representative IF localisation of TGF‐β1 (*top*) and pSMAD3 (*bottom*) in normal and lymphedematous (*labelled LE*) tissues. (B) Quantification of TGF‐β1 (*top*) and pSMAD3 (*bottom*) IF staining areas in tissue sections of patients with unilateral BCRL. Each circle represents an average of three HPF views per patient (*N* = 8). (C) mRNA expression of TGF‐β isoforms and TGF‐βRI comparing normal and lymphedematous limb of patients with unilateral BCRL. Each circle represents an individual patient (*N* = 14). (D) Representative Western blot of TGF‐β isoforms, pSMAD3 and tSMAD3 in normal and lymphedematous limbs of patients with unilateral BCRL. (E) Quantification of Western blots with relative changes comparing normal and lymphedematous limb of each patient. Each circle represents an average of two separate Western blots per patient (*N* = 8). BCRL, breast cancer‐related lymphedema; TGF‐β1, transforming growth factor‐beta 1; IF, immunofluorescence; LE, lymphedema; HPF, high‐power field; TGF‐βR‐I, transforming growth factor‐beta receptor I

Immunofluorescent staining of tissue biopsies in eight patients showed increased TGF‐β1 expression in the papillary dermis of all patients, a greater than 2‐fold overall increase compared to the normal limb (Figure 1A, upper panel; Figure 1B, upper panel; *p* < .02). The pattern of TGF‐β1 expression correlated to that of p‐SMAD3 expression, which was also increased in all patients (Figure 1A, lower panel; Figure 1B, lower panel; *p* < .001).

To confirm our immunofluorescent staining, we analysed mRNA expression of TGF‐β isoforms and downstream signaling molecules in 14 patient samples (Figure 1C and Supplemental Figure S1A). This analysis showed that the expression of TGF‐β1 and TGF‐β3, but not TGF‐β2, was upregulated in the skin of lymphedematous arm as compared to the normal (*p* < .05 for TGF‐β1 and TGF‐β3). TGF‐β1 mRNA expression was increased in 11 of 14 patients; there was either no difference or a minor decrease in TGF‐β1 expression in lymphedematous tissues in three patients. Similarly, the expression of TGF‐β3 was increased in all but one patient. The expression of TGF‐βRI was increased in lymphedematous tissues (increased in all but one patient; *p* < .01), but we found no difference in mRNA expression in other downstream TGF‐β signaling mediators (Supplemental Figure S1A).

We next analysed correlations between disease factors (duration and volume) and patient variables (age, body mass index [BMI], among others) with the expression of TGF‐β1 mRNA (Supplemental Figure S1B). This analysis showed a weak positive correlation between disease duration and TGF‐β1 expression (*R* ^2^ = 0.25, *p* = .051). In contrast, other factors such as patient age, volume differential, history of radiation or BMI were not correlated with TGF‐β1 mRNA expression.

Western blot analysis in eight patients confirmed our histological and mRNA findings demonstrating increased expression of TGF‐β1, pSMAD3 and total SMAD3 (tSMAD3) in all patient samples we analysed (Figure 1D and E). We found no significant differences in the expression of TGF‐β2 or TGF‐β3, and expression of these isoforms showed some variability, with most patients showing increased expression but a few patients demonstrating decreased expression in the lymphedematous tissues.

### 2.2 BCRL increases fibrosis and ECM deposition

**FIGURE 2:**
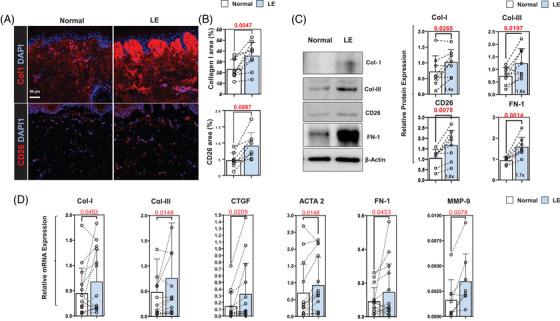
BCRL increases fibrosis and ECM deposition. (A) Representative IF localisation of type 1 collagen (Col1; *top*) and CD26 (*bottom*) in normal and lymphedematous (*labelled LE*) tissues. (B) Quantification of type I collagen (*top*) and CD26 (*bottom*) IF staining areas in tissue sections of patients with unilateral BCRL. Each circle represents an average of three HPF views per patient (*N* = 8). (C) *Left panel*: Representative Western blot of ECM proteins in normal and lymphedematous limbs of patients with unilateral BCRL*. Right panel*: Quantification of ECM proteins. Each circle represents an average of two separate Western blots per patient (*N* = 8). (D) mRNA expression of ECM molecules comparing normal and lymphedematous limb of patients with unilateral BCRL. Each circle represents an individual patient (*N* = 12–14). BCRL, breast cancer‐related lymphedema; ECM, extracellular matrix molecules; IF, immunofluorescence; LE, lymphedema; HPF, high‐power field

Analysis of ECM products in tissue biopsies showed that type I collagen expression was increased in both papillary and reticular dermis of the lymphedematous limb in all patients (Figure 2A, upper panel; Figure 2B, upper panel). The number of CD26^+^ cells, a marker of dermal fibroblasts,^35^, ^36^ was also increased in lymphedematous biopsy samples in all patients (Figure 2A, lower panel; Figure 2B, lower panel). These histological changes correlated with Western blots performed on eight patients, demonstrating increased expression of type I and type III collagens, CD26 and fibronectin‐1 (FN‐1) (Figure 2C). This pattern of increased ECM protein expression was noted in virtually all patients included in our analysis, although two patients had little to no difference in type I collagen protein expression while one patient had slightly decreased type III collagen protein expression.

PCR analysis of patient samples showed some variability in gene expression – particularly in type I collagen, in which we noted modestly decreased type I collagen mRNA expression in three out of 16 patients (Figure 2D). However, the overall analysis showed that lymphedema increases the mRNA expression of type I and type III collagen, connective tissue growth factor (CTGF), ACTA‐2, FN‐1 and MMP‐9. We did not note significant changes in the expression of TIMP‐1, TIMP‐2 and vimentin (Supplemental Figure S2).

### 2.3 Neutralisation of TGF‐β1 decreases lymphedema and inflammation

**FIGURE 3:**
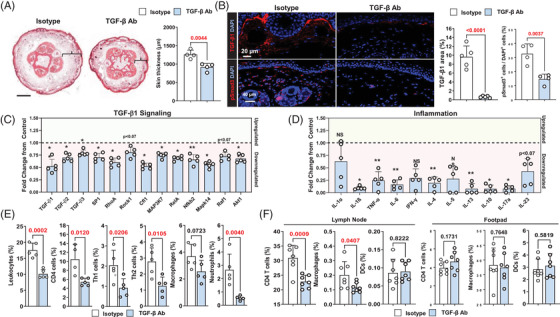
Neutralisation of TGF‐β1 decreases lymphedema and inflammation. (A) *Left panel*: Representative cross‐sectional H&E stain of a mouse tail treated with isotype control or TGF‐β1 neutralising antibody. Brackets show subcutaneous fibroadipose tissues; note decreased subcutaneous thickness in TGF‐β1 neutralising antibody‐treated mice. *Right panel*: Quantification of skin thickness in control or TGF‐β1 neutralising antibody‐treated mice. Each circle represents an average of three HPF views per animal (*N* = 4). (B) *Left panel*: Representative IF localisation of TGF‐β1 (*top*) and pSmad3 (*bottom*) in isotype and TGF‐β1 neutralising antibody‐treated mice. *Right panel*: Quantification of TGF‐β1 area and Smad3+ cells. Each circle represents an average of three HPF views per animal (*N* = 5). (C) mRNA expression of TGF‐β1 isoforms and downstream signaling pathways. Relative change to isotype control‐treated mice is shown. Each circle represents an individual animal (*N* = 5). Genes shown in the green‐ and red‐shaded zones represent upregulated (*green*) and downregulated (*red*) molecules. *p < .05, **p < .01. (D) mRNA expression of inflammatory mediators. Relative change to isotype control‐treated mice is shown. Each circle represents an individual animal (*N* = 5). Genes shown in the green‐ and red‐shaded zones represent upregulated (*green*) and downregulated (*red*) molecules. *p < .05, **p < .01. (E) Quantification of flow cytometry for leukocytes (CD45^+^), CD45^+^/CD4^+^ cells, Th1 (CD45^+^/CD4^+^/CXCR3^+^/CCR5^+^) cells, Th2 (CD45^+^/CD4^+^/CCR4^+^/CCR8^+^) cells, macrophages (CD45^+^/CD11b^+^/F480^+^) and neutrophils (CD45^+^/CD11b^+^/Ly‐6G^+^). Each circle represents an average of two flow cytometry runs for each animal (*N* = 5). (F) Quantification of CD4 T cells (CD45^+^/CD4^+^), macrophages (CD45^+^/CD11b^+^/F480^+^) and DCs (CD45^+^/CD11c^+^/CD86^+^) in draining lymph nodes and foot pad injection site after injection of LE lysate. Each circle represents an individual animal (*N* = 5). TGF‐β1, transforming growth factor‐beta 1; H&E haematoxylin and eosin; HPF, high‐power field; IF, immunofluorescence; DCs, dendritic cells; LE, lymphedema

To determine how increased TGF‐β1 expression affects to the pathology of lymphedema, we used a mouse tail lymphedema model.^13^, ^23^, ^28^, ^37^, ^38^ Two weeks after surgery, mice were treated with TGF‐β1 monoclonal antibodies or isotype control antibodies for 4 weeks, at which point the animals were sacrificed and analysed. Neutralisation of TGF‐β1 with antibodies decreased tail edema, deposition of fibroadipose tissues and expression of TGF‐β1 and pSmad3 in the skin (Figure 3A and B; *p* < .01). A qPCR analysis of tail tissues showed that neutralisation of TGF‐β1 decreases the expression of all TGF‐β isoforms and TGF‐β downstream signaling molecules (Sp1, RhoA, Cfl1, Map3k7, Mapk14, RelA, Nfκb2 and Akt1) and inflammatory mediators (IL‐1β, TNF‐α, IL6, IL4, IL13, IL10 and IL‐17α; Figure 3C and D). TGF‐β neutralisation had non‐significant effects on the mRNA expression of other TGF‐β signaling molecules, lymphatic genes or lymphangiogenic cytokines (Supplemental Figure S3A and B).

Using flow cytometry, we found that TGF‐β1 neutralisation decreases the percentage of leukocytes, CD4^+^ cells, Th1 cells, Th2 cells and neutrophils in the skin, suggesting that this treatment has broad anti‐inflammatory effects in lymphedema (Figure 3E, Supplemental Figure S3D). The number of macrophages was not significantly decreased in animals treated with anti‐TGF‐β1 antibodies. To identify the source of TGF‐β1 in lymphedematous tissues, we performed flow cytometry to isolate stromal cells, CD11b^+^ and CD4^+^ cells from tail tissues of isotype control antibody‐treated mice and performed qPCR (Supplemental Figure S3C). This analysis demonstrated that the expression of TGF‐β1 was highest in CD11b^+^ cells. CD4^+^ cells also expressed TGF‐β1 mRNA but to a lesser degree. Stromal cells (primarily fibroblasts) had the lowest expression of TGF‐β1.

To determine if lymphedema fluid can activate inflammatory responses in draining lymph nodes or local tissues, we harvested tissue lysate from the tails of animals that had tail surgery and were treated with either isotype control or TGF‐β1 neutralising antibodies beginning 2 weeks after surgery for 2 weeks (Figure 3F and Supplemental Figure S3E). Injection of tissue lysate collected from isotype antibody‐treated animals into the foot pad of naive mice did not cause inflammation in the foot pad but increased the percentage of CD4^+^ T cells and macrophages in the draining lymph nodes, while with injection of fluid collected from TGF‐β1 neutralising antibody‐treated mice did not.

### 2.4 TGF‐β1 increases fibroblast ECM expression and increases the stiffness of fibroblasts, LECs and LSMCs

**FIGURE 4:**
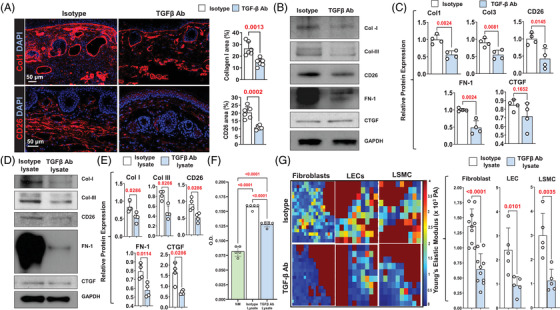
TGF‐β1 increases fibroblast ECM expression and increases the stiffness of fibroblasts, LECs and LSMCs. (A) *Left panel*: Representative IF localisation of type I collagen (Col1) (*top*) and CD26 (*bottom*) in isotype and TGF‐β1 neutralising antibody‐treated mice. *Right panel*: Quantification of type I collagen (*top*) and CD26 (*bottom*) IF staining areas in tissue sections. Each circle represents an average of three HPF views per animal (*N* = 4). (B) Representative Western blot of ECM proteins in mouse tails of animals treated with isotype control or TGF‐β1 neutralising antibodies. (C) Quantification of Western blots of ECM proteins with relative changes comparing isotype control and TGF‐β1 neutralising antibody‐treated mice. Each circle represents an average of two separate Western blots per animal (*N* = 4). (D) Representative Western blot of ECM proteins in NIH3T3 fibroblasts stimulated with LE lysate containing isotype control or TGF‐β1 neutralising antibodies. (E) Quantification of Western blot of ECM proteins in NIH3T3 fibroblasts stimulated with LE lysate containing isotype control or TGF‐β1 neutralising antibodies. Each circle represents an average of four separate Western blots per group. (F) Proliferation of NIH3T3 fibroblasts 24 and 72 h after stimulation with normal media (*labelled NM*) or media supplemented with LE lysate containing isotype control or TGF‐β1 neutralising antibodies. (G) *Left panel*: Representative atomic force microscopy heatmaps of fibroblasts, LECs and LSMCs, stimulated with LE lysate containing isotype control or TGF‐β1 neutralising antibodies*. Right panel*: Quantification of Young's elastic modulus in fibroblasts, LECs and LSMCs stimulated with LE lysate containing isotype control or TGF‐β1 neutralising antibodies. TGF‐β1, transforming growth factor‐beta 1; ECM, extracellular matrix molecules; LECs, lymphatic endothelial cells; LSMCs, lymphatic smooth muscle cells; IF, immunofluorescence; HPF, high‐power field; LE, lymphedema

Immunofluorescent localisation of type I collagen and CD26 in tails harvested from control mice treated with isotype neutralising antibodies showed similar patterns as our clinical lymphedema specimens (Figure 4A). We noted the accumulation of collagen fibers in the papillary dermis and many CD26^+^ cells in the papillary and reticular dermis. TGF‐β1 neutralisation significantly decreased type I collagen deposition and markedly reduced the number of CD26^+^ cells in the tail skin. These histological changes correlated with expression of ECM proteins as assessed by Western blotting, demonstrating decreased expression of type I and III collagen, CD26, FN‐1 and CTGF in animals treated with TGF‐β1 neutralising antibodies (Figure 4B and C).

We collected tissue lysates from the tails of control mice that had tail skin incision only (control lysate) and experimental mice that had tail surgery (lymphedema [LE] lysate) 2 weeks after surgery and found LE and resulting lymphedema increased the expression of TGF‐β1 and VEGF‐C protein (Supplemental Figure S4A). Western blot analysis of mouse fibroblasts treated with LE lysate supplemented with TGF‐β1 neutralising antibodies showed that loss of TGF‐β1 activity decreased expression of ECM molecules as compared with fibroblasts treated with LE lysate supplemented with isotype (control) antibodies (Figure 4D and E). Fibroblasts treated with LE lysate/TGF‐β1 neutralising antibodies also had decreased proliferation compared to cells treated with LE lysate/isotype antibodies (Figure 4F). Interestingly, fibroblasts treated with LE lysate/TGF‐β1 neutralising antibodies proliferated modestly more rapidly than fibroblasts in media alone, suggesting that other factors in lymphedematous tissues can also modulate fibroblast proliferation. To determine whether canonical or non‐canonical pathways regulated the effects of LE Lysate on fibroblast proliferation, fibroblasts were stimulated with LE lysate, LE lysate and a p38 small‐molecule inhibitor or LE lysate with a Smad inhibitor for 72 h (Supplemental Figure S4B). Both p38 and Smad3 inhibition decreased fibroblast proliferation in response to LE lysate; however, Smad3 inhibition was much more effective. Finally, using atomic force microscopy, we found that fibroblasts, LECs and LSMCs treated with LE lysate/TGF‐β1 neutralising antibodies had decreased stiffness compared with cells treated with LE lysate/isotype antibodies (Figure 4G).

### 2.5 Inhibition of TGF‐β1 signaling in LECs increases lymphangiogenesis but does not improve lymphedema

TGF‐β1 has anti‐lymphangiogenic effects in many physiologic and pathologic settings.^26^, ^32^ Some studies have suggested that the beneficial effects of TGF‐β blockade on lymphedema may be related to improved lymphangiogenesis and formation of collateral lymphatics.^13^, ^34^ To directly assess this hypothesis, we created lymphatic‐specific Cre‐lox transgenic mice by mating Flt4 (VEGFR3) Cre animals with floxed mice that express a dominant‐negative TGF‐βRII molecule that binds all three TGF‐β isoforms but does not activate intracellular signaling pathways. Cre activation with tamoxifen in homozygous offsprings (LEC^DN‐TBRII^) results in expression of the TGF‐βRII molecule in all cells that express FLT4 (primarily LECs).^39^

**FIGURE 5:**
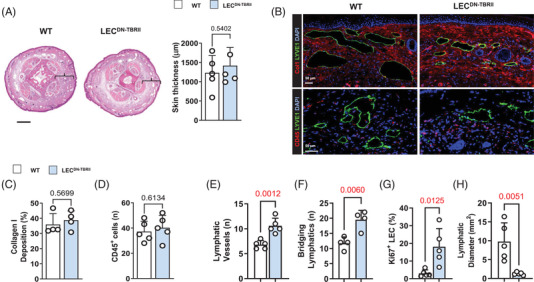
Inhibition of TGF‐β1 signaling in LECs increases lymphangiogenesis but does not improve lymphedema. (A) *Left panel*: Representative cross‐sectional H&E stain of WT or LEC^DN‐TBRII^ mouse tails*. Right panel*: Quantification of skin thickness in WT and LEC^DN‐TBRII^ mouse tails. Each circle represents an average of three HPF views per animal (*N* = 5). Bar = 500 μm. (B) Representative IF localisation of type I collagen (Col1) (*top*) and CD45 (*bottom*) in WT and LEC^DN‐TBRII^ mouse tails. (C) Quantification of type I collagen deposition in WT and LEC^DN‐TBRII^ mouse tails. Each circle represents an average of three HPF views per animal (*N* = 4). (D) Quantification of CD45^+^ cells in WT and LEC^DN‐TBRII^ mouse tails. Each circle represents an average of three HPF views per animal (*N* = 5). (E) Quantification of lymphatic vessels in WT and LEC^DN‐TBRII^ mouse tails. Each circle represents the average of three HPF views per animal (*N* = 6). (F) Quantification of bridging lymphatic channels in WT and LEC^DN‐TBRII^ mouse tails. Each circle represents an average of three HPF views per animal (*N* = 4). (G) Quantification of Ki67^+^ LECs in lymphatic vessels of WT and LEC^DN‐TBRII^ mouse tails. Each circle represents an average of three HPF views per animal (*N* = 5). (H) Lymphatic diameter in WT and LEC^DN‐TBRII^ mouse tails. Each circle represents the average of four lymphatic vessels per animal (*N* = 5). TGF‐β1, transforming growth factor‐beta 1; LECs, lymphatic endothelial cells; H&E haematoxylin and eosin; WT, wild‐type; HPF, high‐power field; IF, immunofluorescence

Analysis of lymphatic vessels in LEC^DN‐TBRII^ showed loss of pSmad3 expression in LECs compared to wild‐type controls (Supplemental Figure S5A). Consistent with the anti‐lymphangiogenic roles of TGF‐β,^26^, ^32^ our inflammatory corneal lymphangiogenesis assay revealed that LEC^DN‐TBRII^ mice had increased corneal lymphangiogenesis and lymphatic branching compared to wild‐type mice (Supplemental Figure S5B and C). Surprisingly, a comparison of LEC^DN‐TBRII^ and wild‐type mouse tails 6 weeks after tail skin and lymphatic excision did not show significant differences in tail swelling, adipose or type I collagen deposition (Figure 5A and B, top panel, and Figure 5C). We also did not find any differences in TGF‐β1 protein expression when comparing the control and LEC^DN‐TBRII^ mice (Supplemental Figure S5D). Loss of TGF‐β signaling in LECs also had no effect on the infiltration of leukocytes (Figure 5B, bottom panel, and Figure 5D). However, we found that LEC^DN‐TBRII^ had an increased number of lymphatic vessels in their tissues and that these lymphatic vessels more commonly bridged the areas of surgical lymphatic excision (Figure 5E and F and Supplemental Figure S5E). In addition, lymphatic vessels in LEC^DN‐TBRII^ were more likely to display a proliferative phenotype, as evidenced by Ki67 staining, and had reduced lymphatic vessel diameters (Figure 5G and H). LYVE‐1^+^ structures in these tissues were not only tubular in shape but also stained positively for VEGFR‐3 and podoplanin, confirming that these are lymphatics rather than individual macrophages (Supplemental Figure S5F). These findings suggest that loss of TGF‐β signaling in LECs increases lymphangiogenesis but has no overall effect on lymphedema or fibrosis.

### 2.6 Topical PFD decreases pathology of lymphedema

**FIGURE 6:**
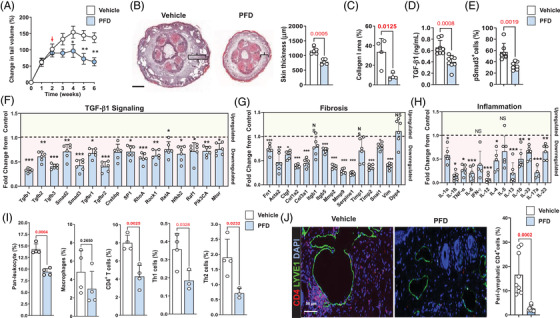
Topical PFD decreases the pathology of lymphedema. (A) Change in tail volume over time in mice treated with vehicle or PFD. Each circle is an average of duplicate measurements from each mouse (*N* = 9 animals per group). Results are presented as mean ± SEM (*p < .05, **p < .01). Statistical comparisons are between groups at the same time points. (B) *Left panel*: Representative cross‐sectional H&E stain of vehicle and PFD‐treated mouse tails. Brackets show subcutaneous fibroadipose tissue deposition. *Right panel*: Quantification of skin thickness in vehicle and PFD‐treated mouse tails. Each circle represents an average of three HPF views from 5 animals. Bar = 500 μm. (C) Quantification of type I collagen deposition in vehicle and PFD‐treated mouse tails. Each circle represents an average of three HPF views from 4 animals. (D) Quantification of TGF‐ β1 protein in tissues collected from vehicle and PFD‐treated mice. Each circle represents an average of duplicate ELISAs per animal (*N* = 9). (E) Quantification of the number of pSmad3+ cells in vehicle and PFD‐treated mouse tails. Each circle represents an average of cell counts in three HPF views from 7 animals. (F) mRNA expression of TGF‐β1 isoforms and downstream signaling pathways. Relative change to vehicle control‐treated mice is shown. Each circle represents an individual animal (*N* = 6). Genes shown in the green‐ and red‐shaded zones represent upregulated (*green*) and downregulated (*red*) molecules. *p < .05, **p < .01, ***p < .001. (G) mRNA expression of fibrosis and ECM genes. Relative change to vehicle control‐treated mice is shown. Each circle represents an individual animal (*N* = 6). Genes shown in the green‐ and red‐shaded zones represent upregulated (*green*) and downregulated (*red*) molecules. *p < .05, **p < .01, ***p < .001. (H) mRNA expression of inflammatory cytokines. Relative change to vehicle control‐treated mice is shown. Each circle represents an individual animal (*N* = 6). Genes shown in the green‐ and red‐shaded zones represent upregulated (*green*) and downregulated (*red*) molecules. *p < .05, **p < .01, ***p < .001. (I) Quantification of flow cytometry for leukocytes (CD45^+^), macrophages (CD45^+^/CD11b^+^/F480^+^), CD45^+^/CD4^+^ cells, Th1 (CD45^+^/CD4^+^/CXCR3^+^/CCR5^+^) cells and Th2 (CD45^+^/CD4^+^/CCR4^+^/CCR8^+^) cells. Each circle represents an average of two flow cytometry runs per animal (*N* = 4). (J) *Left panel*: Representative IF localisation of CD4 (*red*) and LYVE‐1 (*green*) in vehicle and PFD‐treated mouse tails. *Right panel*: Quantification of perilymphatic CD4^+^ cells. Each circle represents an average of three HPF views from 9 animals. PFD, pirfenidone; SEM, standard error of the mean; H&E haematoxylin and eosin; HPF, high‐power field; TGF‐β1, transforming growth factor‐beta 1; ELISA, enzyme‐linked immunosorbent assay; ECM, extracellular matrix molecules; IF, immunofluorescence

Long‐term systemic TGF‐β1 inhibition is not clinically possible because these treatments can cause significant immune disturbances and side effects.^40^, ^41^, ^42^ PFD is a small‐molecule inhibitor that is FDA‐approved for the treatment of idiopathic pulmonary fibrosis, and its mechanism of action is thought to include inhibition of TGF‐β1 activity.^43^ Therefore, to test the effect of PFD on lymphedema in our mouse tail model, animals underwent tail surgery and were treated topically with petrolatum (control) or PFD mixed in petrolatum once daily every day beginning at 2 weeks after surgery for 4 weeks. Topical PFD significantly and rapidly decreased tail lymphedema and fibroadipose tissue deposition in our mouse model (Figure 6A and B and Supplemental Figure S6A). PFD‐treated mice had decreased type I collagen staining, decreased tissue TGF‐β1 protein expression and decreased number of pSmad3^+^ cells in tissues analysed with immunofluorescent staining (Figure 6C–E and Supplemental Figure S6B–D).

PFD decreased the mRNA expression of TGF‐β1 signaling molecules, fibrotic genes and inflammatory genes (Figure 6F–H, and Supplemental Figure S6E). Flow cytometry demonstrated that topical PFD decreased infiltration of leukocytes, CD4^+^ T cells, Th1 cells and Th2 cells but did not alter the number of infiltrating macrophages (Figure 6I). These findings were corroborated by analysis of protein expression in tail tissues using enzyme‐linked immunosorbent assay (ELISA) demonstrating decreased expression of IFN‐γ and IL‐13 (Supplemental Figure S6F). Interestingly, we found that PFD treatment had no effect on VEGF‐C expression but modestly decreased VEGF‐A expression (Supplemental Figure S6F).

Consistent with our previous reports,^23^, ^29^ we found that CD4^+^ cells clustered around the lymphatic vessels of vehicle‐treated mice (Figure 6J). This phenotype was significantly reduced in PFD‐treated animals. In contrast, we found no perilymphatic accumulation of macrophages after PFD treatment (Supplemental Figure S6G).

### 2.7 Topical PFD improves lymphatic function

**FIGURE 7:**
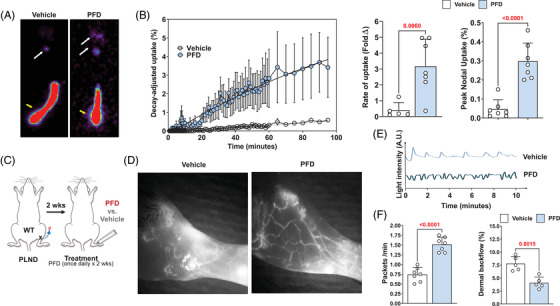
Topical PFD improves lymphatic function. (A) Representative heat map of Tc^99^ uptake in the sacral lymph nodes of vehicle and PFD‐treated mouse tails. (B) *Left panel*: Quantification of decay adjusted Tc^99^ uptake in the sacral lymph nodes of vehicle and PFD‐treated mouse tails. The rate of Tc^99^ uptake (*centre panel*) and peak nodal uptake (*right panel*) are also shown. Each circle represents an average of three measurements per animal at each time point (*N* = 7 animals/group). (C) Experimental plan for PLND and analysis of lymphatic pumping/dermal backflow. (D) Representative ICG image of vehicle and PFD‐treated mice. Note increased collateral vessel formation and decreased dermal backflow (white fluorescence) in PFD‐treated animals. (E) Representative plot showing changes in light intensity in collecting lymphatic vessels of vehicle and PFD‐treated mice. (F) *Left panel*: Quantification of collecting lymphatic pumping (packets/min) in vehicle and PFD‐treated mice. *Right panel*: Quantification of dermal backflow. Each circle represents an average of two measurements per animal (*N* = 5). PFD, pirfenidone; PLND, popliteal lymph node dissections; ICG, indocyanine green

To determine the functional effects of PFD after lymphatic injury, we analysed Tc^99^ uptake in the sacral nodes of animals that had tail surgery and were treated either with vehicle control or topical PFD for 4 weeks. PFD treatment increased lymph node uptake of Tc,^99^ increasing both the slope (i.e. rate) and the total nodal uptake (Figure 7A and B). To confirm these findings and determine how PFD modulates lymphatic pumping and collecting vessels leakiness, we performed popliteal lymph node dissections (PLND) on wild‐type mice, and after a 2‐week recovery period, we treated the animals with either vehicle or topical PFD once daily for 2 weeks (Figure 7C). Analysis of lymphatics with indocyanine green (ICG) lymphangiography revealed that PFD treatment increased the number of collateral lymphatics in the hindlimb, increased the pumping frequency of lymphatic collectors and decreased accumulation of ICG dye in the dermis (i.e. dermal backflow; Figure 7D–F). These changes correlated with decreased numbers of iNOS^+^ cells in the tissues of PFD‐treated animals (Supplemental Figure S7A).

To compare the efficacy of antibody‐mediated TGF‐β1 neutralisation with PFD, we treated mice with PLND as outlined above and then treated them with TGF‐β1 monoclonal antibody, topical PFD or a combination of TGF‐β1 antibody and topical PFD and analysed dermal backflow and lymphatic pumping (Supplemental Figure S7B–E). We found that topical PFD was as effective as TGF‐β1 neutralisation in decreasing dermal back flow and increasing lymphatic pumping. The combination of these two treatments did not further improve lymphatic function.

## 3 DISCUSSION

### 3.1 Lymphedema, fibrosis and TGF‐β1

TGF‐β is a growth factor with three isoforms that play essential roles in various settings, including development, tissue repair, immune responses, fibrosis and cancer.^44^, ^45^ Activated TGF‐β isoforms bind transmembrane TGF‐βR complexes formed by TGF‐βRI and TGF‐βRII, resulting in phosphorylation of TGF‐βRI.^46^ In the canonical TGF‐β signaling pathway, phosphorylated TGF‐βRI activates intracellular signaling by phosphorylating SMAD2 and SMAD3, which form complexes with Smad4 and translocate to the nucleus to regulate transcription. Non‐canonical pathways have also been described and mediate signaling via other pathways, including MAPK‐ERK,^47^ NFkΒ, PI3K‐AKT‐mTOR, RAF‐MEK1,2, ERK1/2, Rho/Rho‐associated kinase.^48^

The expression of TGF‐β1 has been implicated in the pathophysiology of fibrosis in many organ systems, including skin, liver, lung, kidney, heart, bone marrow and pancreas, among others.^49^ TGF‐β1 promotes fibrosis by directly regulating activation, proliferation, migration and production of ECM molecules, such as fibronectin and collagen types I, III and IV.^45^, ^49^ TGF‐β1 single nucleotide polymorphisms (SNPs) are associated with increased susceptibility to radiation‐induced fibrosis and increase the risk of fibrotic lung disease and graft fibrosis after lung transplantation in a subset of patients.^50^, ^51^

Several lines of evidence support the hypothesis that TGF‐β1 also plays an important role in the pathophysiology of lymphedema. Sano et al. showed that increased expression of TGF‐β1 and tissue fibrosis in a rat model of lymphedema and in a small cohort (five patients) with stage II lymphedema.^33^ Another study showed that EW‐7197, a TGF‐βRI receptor kinase inhibitor, decreased the severity of lymphedema and increased lymphangiogenesis when administered to a mouse tail model.^34^ In a previous study, we used a small‐molecule inhibitor of TGF‐β1 in a mouse tail model and showed that radiation therapy inhibits with lymphatic function by TGF‐β1‐related tissue fibrosis.^52^ In other studies, we showed that TGF‐β1 is a negative regulator for the regeneration of lymphatic vessels in wound healing and lymphedema.^13^, ^26^ Our current study expands on these findings by using a bigger number of clinical samples collected from women with unilateral BCRL, thus validating animal model findings. In addition, unlike previous studies in which TGF‐β1 expression was localised using immunohistochemistry or in vitro studies, we analysed the expression of this growth factor and its downstream mediators using qPCR and Western blotting, thus increasing the scientific rigor of previous findings. We also correlated clinical and patient‐related factors to TGF‐β1 expression and found only a weak correlation with the duration of the disease but not with changes in arm volume, patient age or BMI.

We found that LE lysate is highly enriched in TGF‐β1, increases proliferation of fibroblasts in vitro and increases expression of ECM molecules. Importantly, this response is attenuated when cells are treated with TGF‐β1 neutralising antibodies. These findings provide a cellular mechanism for our observation which is the number of CD26^+^ cells is increased significantly in lymphedematous tissues of patients with BCRL and that treatment with TGF‐β1 neutralising antibodies decreases the number of these cells in the mouse tail model of lymphedema. This is important because CD26^+^ fibroblasts are highly proliferative and primarily responsible for connective tissue deposition in wound healing and pathologic conditions such as radiation‐induced fibrosis, keloids and cancer stroma fibrosis.^35^, ^36^, ^53^

### 3.2 The relative contribution of TGF‐β isoforms to fibrosis

All three TGF‐β isoforms increase ECM production and promote fibroblast differentiation in vitro.^54^, ^55^, ^56^ However, the in vivo effects of different TGF‐β isoforms are controversial and contradictory. Some studies have suggested that TGF‐β3 may elicit similar fibrotic responses as TGF‐β1,^57^ while others have shown that TGF‐β3 may be less potent or even play an antifibrotic role.^58^, ^59^ The relative role of TGF‐β2 is even less clear; however, the low binding affinity of TGF‐β2 for TGF‐βRII suggests that this isoform is not as important as TGF‐β1 or TGF‐β3.^60^ In the current study, we noted an increase in TGF‐β1 expression using histology, Western blotting and qPCR in almost every patient and in our mouse model. In contrast, changes in TGF‐β2 and TGF‐β3 were more modest and inconsistent. We found that TGF‐β1 blockade also decreased the mRNA expression of non‐canonical signaling pathways, including RhoA, MAPK, Akt and NFκB, suggesting that both canonical and non‐canonical signaling pathways regulate responses to increased TGF‐β1 expression in lymphedema. It is difficult, however, to determine the precise role of each isoform in vivo. Using isoform‐specific TGF‐β transgenic mice is not possible in most studies because TGF‐β1, 2 and 3 knockout mice have significant immunologic abnormalities and die shortly after birth.^61^, ^62^, ^63^, ^64^ Similarly, our in vitro data suggest that activation of Smad by TGF‐β1 regulates fibroblast proliferation since inhibition of Smad signaling, and to a lesser extent p38 pathway, decreased cellular proliferation in vitro (Supplemental Figure S4C). Thus, it is possible that due to its relative abundance, TGF‐β1 is the most important isoform in the pathophysiology of lymphedema, but additional study is required.

### 3.3 How does fibrosis inhibit lymphatic function?

Our study sheds some light on the cellular mechanisms that translate fibrosis to lymphatic dysfunction and lymphedema. We found that TGF‐β1 in interstitial fluid from lymphedematous tissues increases fibroblast production of ECM molecules and increases fibroblast stiffness. Importantly, we found that TGF‐β1 in LE lysate markedly increases the expression of FN‐1. This is important because FN‐1 affects ECM stiffness and incorporates into the ECM through specific binding domains in heparin, fibrin and collagen.^65^ FN‐1 also regulates collagen fibrillation.^66^ ECM and fibroblast stiffness regulate fibroblast proliferation and differentiation and are important contributors to the pathology of fibrotic disorders and tumor microenvironment.^65^, ^67^ These changes also appear to impair lymphatic function and lymphatic regeneration in secondary lymphedema.

Another interesting finding of our study is that TGF‐β1 expression caused LEC and LSMC stiffness, lymphatic leakiness, impaired collecting vessel pumping and infiltration of iNOS^+^ cells. Thus, while the direct effects of TGF‐β1 on LECs do not appear to be the main mechanism by which this growth factor impairs lymphatic function, changes in the ECM, cellular stiffness and the cell microenvironment may indirectly regulate lymphatic drainage. This hypothesis is supported by that chronic inflammation‐induced blood vascular stiffness is a major cause of cardiovascular pathology by increasing the stiffness of endothelial cells, matrix, fibroblasts and vascular smooth muscle cells.^68^ The ECM provides chemical and mechanical stimuli that regulate new blood vessel formation, endothelial network assembly and differentiation.^69^, ^70^ Thus, while rigid substrates increase cell spreading by promoting cell‐substrate adhesions,^71^ soft matrices promote cellular aggregation and network formation by promoting cell–cell adhesion.^72^ A recent study used atomic force microscopy to show that LECs, like blood endothelial cells, are sensitive to gradients of matrix stiffness.^73^ During embryogenesis, exposure of LEC progenitors arising from the cardinal vein to the soft matrix of embryonic tissues activates the transcription factor GATA2, promoting cellular migration, increasing sensitivity to VEGF‐C, and mediates lymphatic vessel formation. Thus, increased tissue stiffness in lymphedematous tissues may promote LEC proliferation but prevent successful network assembly and inhibit lymphatic function.

### 3.4 TGF‐β regulation of lymphangiogenesis

TGF‐β1 is a potent anti‐lymphangiogenic growth factor in many settings.^26^, ^32^ Previous studies have shown that inhibition of TGF‐β1 after lymphatic injury increases lymphangiogenesis and formation of collateral vessels, suggesting that these effects modulate the beneficial effects of TGF‐β1 blockade.^13^, ^26^, ^33^, ^34^ This hypothesis is supported by the efficacy of lymphangiogenic growth factor treatment in animal models of lymphedema and has led to clinical trials using this approach.^14^, ^15^, ^16^, ^17^, ^18^ Our study is the first to show that the lymphangiogenic effects of TGF‐β1 blockade may not be as important as the effects of these treatments on fibrosis or inflammatory responses. This concept is directly supported by our studies with transgenic mice that have decreased LEC responsiveness to TGF‐β1, in which we found increased lymphangiogenesis but no protection from swelling, fibrosis and inflammation compared with controls. Our study is also supported by previous papers showing that the expression of lymphangiogenic growth factors and the number of LECs is *increased* in lymphedematous tissues, suggesting that lymphedema is not a deficiency of lymphangiogenesis or lymphangiogenic growth factors.^74^, ^75^, ^76^ Indeed, animals that overexpress VEGF‐C have more severe lymphedema and increased lymphatic leakiness.^75^ Taken in this context, our findings suggest that antifibrotic or anti‐inflammatory treatments may be more effective treatment options for lymphedema as compared with efforts aimed at delivering supraphysiologic doses of VEGF‐C.

### 3.5 TGF‐β1 and inflammatory responses

TGF‐β1 plays a crucial role in suppressing autoreactive T cells and peripheral immune tolerance^77^ and is necessary for the proliferation and differentiation of T regulatory cells.^78^, ^79^, ^80^, ^81^ TGF‐β1 also regulates survival and activation of naïve CD4^+^ and CD8^+^ T cells in some contexts, usually by suppressing activation of Th1 and Th2 cells.^82^ In our study, we noted that the number of Th1 and Th2 cells was decreased with TGF‐β1 inhibition or with topical PFD treatment. This seemingly paradoxical response (i.e. increased TGF‐β1 would be expected to suppress Th1/Th2 activation and proliferation) may be an indirect effect of our treatment and related to improvements in the lymphatic clearance of immune cells rather than direct effects of TGF‐β inhibition. This hypothesis is supported by our studies demonstrating that treatment with PFD or TGF‐β1 neutralising antibodies increase lymphatic pumping and transport function. Nevertheless, decreasing Th2 inflammatory cell infiltration may be an important mechanism by which TGF‐β1 blockade improves lymphatic function since Th2‐derived cytokines are key regulators of fibrosis, lymphatic leakiness, impaired collecting vessel pumping and formation of collaterals.^23^, ^37^, ^74^, ^83^, ^84^

Previous studies have suggested that macrophages are key regulators of fibrotic responses by producing proteases that regulate ECM modeling and producing pro‐inflammatory cytokines, including TGF‐β1.^85^ TGF‐β1 is also a potent mitogen and chemoattractant for macrophages. In the current study, we did not find significant changes in the number of macrophages after TGF‐β1 inhibition, suggesting that these cells may not be as important as other inflammatory cell types in chronic lymphedema or that other mechanisms regulate macrophage infiltration in this setting. This hypothesis is supported by our previous studies showing that macrophages have a complicated role in the pathophysiology of lymphedema. In the subacute period following lymphatic injury, the depletion of macrophages decreases lymphatic regeneration, thereby increasing fibrosis and tissue swelling; in contrast, late depletion of these cells does not affect lymphatic vessel counts but results in increased ECM accumulation.^86^, ^87^ Thus, the effects of TGF‐β1 on macrophages in our study may reflect the time and context‐dependent changes.

### 3.6 PFD is an effective treatment for lymphedema

We found that topical PFD is highly effective in treating lymphedema in our mouse model. PFD is also effective for treating other fibrotic disorders, including pulmonary fibrosis, allergen‐induced airway remodeling, cardiac fibrosis, renal fibrosis, systemic sclerosis, keloids and hepatic fibrosis.^43^, ^88^, ^89^, ^90^, ^91^, ^92^, ^93^, ^94^, ^95^ Consistent with previous studies, we found that PFD decreased fibrosis, decreased activation of TGF‐β/downstream signaling, decreased inflammatory cell infiltration and decreased expression of inflammatory cytokines.^43^, ^91^, ^96^, ^97^, ^98^, ^99^ Interestingly, PFD did not alter VEGF‐C expression and modestly decreased the expression of VEGF‐A, suggesting that improvements in lymphatic function were not related to increased lymphangiogenic cytokine activity. Treatment with PFD also significantly improved lymphatic function by increasing lymphatic collecting vessel pumping and collateral vessels formation and decreasing lymphatic leakiness. These effects collectively increased interstitial fluid preload and decreased afterload. This is important because changes in preload and afterload on isolated lymphatic vessels significantly affect lymphatic vessel contractility.^100^, ^101^ Treatment with PFD also decreased infiltration of iNOS^+^ cells. This is important because expression of iNOS from inflammatory cells decreases the eNOS gradients and impairs lymphatic collecting vessel pumping.^102^, ^103^, ^104^ The addition of TGF‐β1 antibody treatment to PFD‐treated mice did not further improve lymphatic function, suggesting that the PFD treatment maximally inhibited TGF‐β1 activity in our model. In contrast to TGF‐β1 neutralising antibody treatment, PFD had mixed effects on the expression of canonical and non‐canonical TGF‐β1 signaling molecules. PFD decreased expression of RhoA, Rock1, NFκB, Pi3kCA and Mtor but had no effects on MAPK and Akt1, suggesting that these latter pathways are less important in the pathophysiology of lymphedema.

### 3.7 Limitations

Our study has some limitations. Most importantly, although this is the largest study to date to analyse changes in TGF‐β1 expression in clinical samples, more studies are needed to analyse clinical and patient factors that may regulate the expression of TGF‐β1 in lymphedema. Although our study suggests that TGF‐β1 is the dominant isoform in lymphedema, an analysis of the inhibition of different isoforms may also be interesting. Our mouse model closely correlates with the histological findings of lymphedema, but, as with all animal models, may not reflect the whole picture. This is a particular problem in lymphedema, as evidenced by a large number of proposed animal models.^105^, ^106^, ^107^ Nevertheless, corroborating the results of our animal studies with clinical samples is important and useful for validating our findings.

## 4 CONCLUSIONS

TGF‐β1 expression is increased in lymphedema and regulates fibrosis, formation of collateral lymphatics and inflammation. The direct effects of TGF‐β1 on LECs are less important than the fibrotic and inflammatory manifestations of this growth factor. Topical treatment with PFD is highly effective in treating lymphedema in a mouse model of the disease.

## 5 MATERIALS AND METHODS

### 5.1 Clinical lymphedema biopsy specimens

All procedures were approved by the Institutional Review Board (IRB) at Memorial Sloan Kettering Cancer Center (MSK) (IRB protocol 17–377); all patients provided written informed consent. Women with unilateral upper extremity BCRL were identified in our lymphedema clinic and screened for eligibility for harvesting of biopsy specimens. Inclusion criteria included age between 21 and 75, unilateral axillary surgery, stage I–III lymphedema (volume differential of >10% with the normal limb or L‐Dex [ImpediMed, Carlsbad, CA, USA] measurements above 7.5 units). Exclusion criteria included pregnancy or lactating women, recent (within 3 months) history of lymphedematous limb infection, chemotherapy, treatment with steroids or other immunosuppressive agents and active cancer or breast cancer metastasis. We harvested 5‐mm full‐thickness skin biopsies from the volar surface of the normal and lymphedematous limb at a point located 5–10 cm below the elbow crease. Surgery was performed under sterile conditions with local anaesthesia. Patients were treated with a dose of antibiotics (cephalexin 1000 mg or clindamycin 600 mg if penicillin‐allergic) 30–60 min before the procedure.

### 5.2 Animals

All studies were approved by the Institutional Animal Care and Use Committee (IACUC) at MSK under protocol (06‐08‐018). The MSK IACUC adheres to the National Institutes of Health Public Health Service Policy on Humane Care and Use of Laboratory Animals and operates in accordance with the Animal Welfare Act and the Health Research Extension Act of 1985 per the IACUC‐approved protocol.

Adult (10‐ to 14‐week‐old) C57BL/6J were used for all treatment studies. To investigate the direct role of TGF‐β1 on LECs, we developed an inducible transgenic mouse with a dominant‐negative TGF‐β receptor on LECs. Thus, in contrast to systemic inhibition of TGF‐ β1, this animal model enables us to selectively inhibit TGF‐β1 signaling in LECs. This was accomplished by crossing FLT4‐CreER^T2^ mice (a gift from Dr. Sagrario Ortega), in which the FLT4 promoter of VEGFR3 is under the control of estrogen receptor type 2 and is highly expressed by all LECs in adult mice,^108^ with B6;129‐*Tgfbr2tm1Karl*/J mice (The Jackson Laboratory) that possess *LoxP* sites flanking exon 4 of TβRII. The resultant homozygous mice (LEC^DN‐TBRII^) express the dominant‐negative TGF‐βRII molecule, which binds TGF‐β (all isoforms) but does not activate intracellular signaling cascades.^109^ Cre expression was induced using tamoxifen (300 mg/kg/day intraperitoneal injections for 5 days). Age‐matched transgenic mice that were not treated with tamoxifen were used as controls for all studies.

### 5.3 Surgical models of lymphedema

We used two different previously described mouse models of lymphedema.^110^ In the tail surgery model, both the superficial and deep lymphatic vasculature were ligated through a 2‐mm circumferential excision of the tail skin 2 cm far from the base.^23^, ^111^ For analysis of lymphatic pumping, PLND was performed to remove the lymph node and nearby fat tissue which has efferent and afferent lymphatic vessels.^110^, ^112^ Animals were euthanised by carbon dioxide asphyxiation as recommended by the American Veterinary Medical Association.

### 5.4 Histology and immunofluorescence

Histological and immunofluorescence analysis was performed using our previously published techniques.^13^, ^74^, ^113^ When indicated, clinical biopsy specimens, corneas, tails and hindlimbs were harvested and fixed in 4% paraformaldehyde (Sigma‐Aldrich, St. Louis, MO, USA) overnight. Corneal whole‐mount staining was performed after digestion with proteinase K in a 10 mM Tris‐HCl buffer (Sigma‐Aldrich) solution. Tissues were washed in 100% methanol and blocked by using donkey serum (Sigma‐Aldrich) in 2% BSA before incubation with primary antibodies (Table 2) for 16 h at 4°C. Antibody staining was visualised with fluorescent‐labelled secondary antibody conjugates (Invitrogen, Burlingame, CA, USA) for 5 h and 4,6‐diamidino‐2‐phenylindole (DAPI; #D1306, Molecular Probes/Invitrogen, Eugene, OR, USA) for 10 min. Imaging was performed with a Leica SP5‐U confocal microscope (Leica Microsystems, Buffalo Grove, IL, USA), and quantification was conducted with Imaris software (Bitplane, Zurich, Switzerland).

**TABLE 2:** The list of antibodies

Target	Origin	Ratio	Cat. No.	Company	City, Country
LYVE‐1	Goat	1:400	#2125	R&D system	Minneapolis, MN, USA
CD31	Rat	1:300	#553370	BD Biosciences	San Jose, CA, USA
CD45	Rat	1:100	#MAB114	R&D system	Minneapolis, MN, USA
CD26	Goat	1:400	#AF954	R&D system	Minneapolis, MN, USA
CD31	Armenian hamster	1:1000	#MAB1398Z	MilliporeSigma	Burlington, MA, USA
iNOS/NOS type II	Mouse	1:400	#610329	BD Biosciences	San Jose, CA, USA
Collagen I	Rabbit	1:100	#ab34710	Abcam	Waltham, MA, USA
pSmad3	Rabbit	1:400	#ab52903	Abcam	Waltham, MA, USA
TGF‐β1	Rabbit	1:300	#ab170874	Abcam	Waltham, MA, USA
Ki67	Rabbit	1:200	#ab16667	Abcam	Waltham, MA, USA

Tails and hindlimbs were decalcified by following previously described protocol.^23^ For immunofluorescent staining, the rehydrated sections underwent antigen recovery with sodium citrate buffer (Sigma‐Aldrich) and quenching of endogenous peroxidase activity. Anti‐mouse primary antibodies used for this study are described in Table 2.

H&E and immunofluorescence slides were evaluated with brightfield or fluorescent microscopy and scanned using a Mirax slide scanner (Zeiss). Staining was visualised using Pannoramic Viewer (3DHISTECH Ltd., Budapest, Hungary). Fibroadipose tissue deposition was quantified in H&E‐stained sections by measuring the width of dermis. Type I collagen deposition was quantified following previously described protocol.^37^

### 5.5 Western blotting

Western blot and analysis were performed using our previously published techniques.^23^ Membranes were stained with antibodies against TGF‐β1 (Abcam, ab170874, 1:500), TGF‐β2 (Thermo Fisher Scientific,  MA537505, 1:500), TGF‐β3 (R&D Systems, AF‐243‐NA, 1:500), phospho‐SMAD3 (Abcam, ab51451, 1:500 dilution), pan‐SMAD3 (Cell Signaling, 8685, 1:1000), collagen I (Thermo Fisher Scientific, MA1‐141 for human; PA1‐26204 for mouse, 1:1000), collagen III (Proteintech, 22734‐1‐AP, 1:1000), CD26 (R&D Systems, AF954 for mouse, 1:1000; Thermo Fisher Scientific, MA532643 for human, 1:1000), fibronectin (Abcam, ab2413, 1:1000), β‐actin (Cell Signaling, 3700s, 1:1000) and GAPDH (MilliporeSigma, MAB374, 1:2000 dilution). Protein expression was quantified with ImageJ software and normalised with housekeeping genes, GAPDH or β‐actin.

### 5.6 PCR

Total RNA was extracted using TRIzol (Invitrogen, Carlsbad, CA, USA) according to the manufacturer's instruction, and complementary DNA (cDNA) was prepared by using Maxima™ H Minus cDNA Synthesis Master Mix (Thermo Scientific, Rockford, IL, USA). Real‐time qPCR (qRT‐PCR; ViiA7; Life Technologies, Carlsbad, CA, USA) was performed in duplicates using predesigned primer sets (Quantitect Primer Assays, Qiagen, Germantown, MD, USA). Relative mRNA expression was analysed normalised to housekeeping genes, β‐actin or GAPDH.

### 5.7 Tail volume measurements

Tail volumes (*V*) were calculated weekly following tail surgery to evaluate the development of lymphedema over time.^26^ Digital calipers were used to measure tail diameter every 1 cm starting at the surgical site going distally towards the tip of the tail. Serial circumferences (*C*) were determined and used to calculate tail volume per the truncated cone formula (*V* = 1/4π [*C*_1_ *C*_2_ + *C*_2_ *C*_3_ + *C*_3_ *C*_4_]).

### 5.8 TGF‐β1 blockade

Mice were treated with intraperitoneal injections of either 5 mg/kg of anti‐mouse TGF‐β1 monoclonal antibody (clone 1D11.16.8; Bio X Cell, West Lebanon, NH, USA)^114^ or non‐specific mouse IgG1 isotype control (clone MOPC‐21; Bio X Cell) diluted in 150 μl of phosphate‐buffered saline (PBS; Mediatech, Inc., Manassas, VA, USA). The injections were administered three times a week for 2 or 4 weeks after PLND or tail excision, respectively.

### 5.9 Analysis of lymphatic function

Lymphatic pumping was analysed using near‐infrared lymphangiography per our previously published methods.^29^ Briefly, 15 μl of 0.15 mg/ml of ICG (Sigma‐Aldrich, St. Louis, MO, USA) was injected intradermally into the ipsilateral hindfoot after induction of anaesthesia. The animals were subsequently awakened and permitted to move freely to allow uptake of ICG into lymphatic vessels. After 20 min, anaesthesia was induced once again, and hindlimb collecting lymphatic vessels were visualised. Images were obtained and analysed using Fiji software (National Institutes of Health, Bethesda, MD, USA). Lymphatic vessel pumping (packet frequency) was quantified as contractions per minute. Lymphoscintigraphy was performed as previously described.^23^, ^26^

### 5.10 Corneal lymphangiogenesis assay

Corneal lymphangiogenesis assay followed previously written protocol.^84^ Briefly, three 11‐0 sutures are placed in the central cornea. The outermost portion of the suture is placed halfway between the limbus and the line outlined by the trephine, while the innermost is equidistant from the trephine line. Animals were sacrificed 14 days later for analysis. ImageJ software (National Institutes of Health, Bethesda, MD, USA) was used to quantify the number of lymphatic vessels branching points.

### 5.11 ELISA

ELISA was performed using our published methods.^29^ The following ELISA kits were used: IFN‐γ (#88‐7314), IL‐13 (#BMS605), TGF‐β1 (#BMS608) and VEGF‐A (#BMS619) from Invitrogen; and VEGF‐C (#028842) from US Biological (Salem, MA, USA). All samples were assessed in triplicate.

### 5.12 Flow cytometry

Flow cytometry was performed to quantify inflammation in the mouse tails after tail surgery.^37^ Briefly, single‐cell suspensions were obtained from a 1‐cm portion of the tail distal to the surgical site using a combination of mechanical dissociation and enzymatic digestion. Cells were stained with anti‐mouse monoclonal antibodies: rat CD45 (30‐F11; #103139), rat CD3 (12A2; #100203), rat CD4 (RM4‐5; #100536), Armenian hamster CXCR3 (CXCR3‐173; #126511), rat CD11b (M1/70; #101207), Armenian hamster CCR4 (2G12; #131211), mouse Ly‐6G (1A8, #127607),), rat CD86 (GL‐1; #105011) and rat CCR8 (SA214G2; #150309), rat F4/80 (BM8; #MF48004), Armenian hamster CD11c (N418; #45‐0114‐82) from BioLegend (San Diego, CA, USA); Armenian hamster CCR5 (7A4; #12‐1951‐82) from eBioscience (San Diego, CA, USA).

For cell sorting, mouse tail skin was harvested 2 weeks after tail surgery and digested into single‐cell suspensions. Three different population (CD45+CD11b+, CD45+CD4+, CD45–) were obtained with BD FACS Aria™III (BD Bioscience).

### 5.13 Analysis of LE lysate effect on local and lymph node inflammation

Tail tissue LE lysate was collected from adult female C57B6 mice that had tail surgery and were treated either with TGF‐β1 monoclonal antibody (TGFβ1 Ab lysate) or non‐specific mouse IgG1 isotype control (Isotype Ab lysate) for 2 weeks beginning 2 weeks after tail surgery. Antibodies were administered once per week. Tail skin and subcutaneous tissues were collected, cut into small pieces and washed with ice‐cold PBS. The tissues were then grinded with RIPA buffer and homogenised. The resultant homogenised fluid was centrifuged for 10 min at 4°C. For fibroblast proliferation studies, cell culture medium (Dulbecco's modified Eagle's medium [DMEM] containing 2 M glutamine and 10% FCS, all from Sigma‐Aldrich) was prepared with 20% per volume of control or LE tissue lysate.

For analysis of local or lymph node inflammatory cell infiltrate, naïve adult female C57B6 mice were with 10 μl of LE lysate injected intradermally into the foot pad. One week after injection, animals were sacrificed and the injection site as well as the popliteal lymph node draining the injection area were harvested and analysed using flow cytometry as described above.

### 5.14 In vitro fibroblast studies and atomic force microscopy

We collected tail tissues from control mice that had tail skin incision only (control lysate) and experimental mice (LE lysate) that had tail surgery, 2 weeks after surgery. Control lysate and LE lysate were created as outlined above using a homogeniser. For fibroblast proliferation studies, cell culture media (DMEM containing 2 M glutamine and 10% FCS, all from Sigma‐Aldrich) was prepared with 20% per volume of control or LE tissue lysate. For TGF‐β1 inhibition, TGF‐β mAb (50 ng/ml) or non‐specific mouse IgG1 isotype control antibody was added to the culture media. To inhibit Smad3 or p38 activation in culture, we supplemented the media with SIS3 HCL (5 μM) or SB 203580 (30 mM; both from APExBIO, Houston, TX, USA), respectively. These molecules are highly specific for Smad3 and P38.^115^, ^116^

An MFP‐3D‐BIO atomic force microscope (Asylum Research, Santa Barbara, CA, USA) was used to examine the stiffness of cells treated with tail lysate from mice treated with isotype or TGF‐β neutralising antibody. To study changes in fibroblasts, NIH3T3 cells were plated with a density of 2.5 × 10^5^ into 50‐mm glass‐bottom dishes and cultured in DMEM containing 10% FBS overnight. The cells were then washed with PBS and incubated in the media containing 20% LE lysate collected from animals treated with either isotype control or TGF‐β1 neutralising antibody for 48 h. In other experiments, we harvested afferent collecting lymphatic vessels leading from Flt4^cre^GFP^floxed^ mice and incubated these tissues in endothelial cell growth medium (ECGM) MV2 complete media containing 20% LE lysate collected from animals treated with either isotype control or TGF‐β1 neutralising antibody for 48 h. The lymphatic vessels were fixed briefly with 2% paraformaldehyde, embedded in optimal cutting temperature media (Tissue‐TEK, Torrance, CA, USA) and sectioned into a 50‐mm glass‐bottom dish. The lymphatic sections were stained with anti‐SMA conjugated with Cy3 for 1 h at 37°C, thus distinguishing between LECs (GFP^+^) and LMCs (Cy3^+^). A NovaScan (Boone, IA, USA) probe with a 5‐μm borosilicate glass bead was used. The Asylum Research Thermal calibration method was used to determine the spring constant (∼0.1 N/m). Each force map sampled a 60 μm × 60 μm region in an 18 × 18 grid under fluid conditions (DMEM containing 10% FBS for fibroblast cell line, NIH3T3 cells; ECGM MV2 media supplemented with 5% FCS for LSMCs). Experimental setting and analysis were followed by previously described protocols.^117^

### 5.15 PFD treatment

A topical formulation of PFD (1% PFD dissolved in Aquaphor^®^; Beiersdorf, Hamburg, Germany) was developed in collaboration with the Research Pharmacy Core Facility at MSK. This dose of PFD was based on previous studies showing effective treatment regimens for various models of fibrosis.^91^, ^118^, ^119^ Control group was treated with Aquaphor^®^ alone. PFD or Aquaphor treatment was initiated 2 weeks after tail surgery. The treatment was applied once daily for 4 weeks to the tail region distal to the zone of lymphatic/skin excision.

### 5.16 Statistical analysis

Statistical analysis was obtained by GraphPad Prism. Normal distribution of the samples was confirmed with the Shapiro–Wilk test. Normally distributed clinical samples were analysed using a matched Student's *t*‐test. Non‐normally distributed samples were analysed using the Wilcoxon matched‐pairs signed‐rank test. Comparison of multiple groups or time points was performed using one‐way or two‐way ANOVA with multiple comparisons using Tukey's multiple comparison test. Correlations between TGF‐β1 gene expression and patient/disease factors were performed using simple linear regression. Preliminary studies were used for power analysis to avoid type II statistical errors. All the data are shown with the mean value ± standard deviation (otherwise it is noted), and *p* value lower than .05 were considered as significant.

## CONFLICT OF INTEREST

Dr. Mehrara is an advisor to PureTech Corporation and recipient of an investigator‐initiated award from PureTech and Regeneron Corp.

## DISCLOSURES

Dr. Mehrara serves as an advisor to PureTech and is the recipient of investigator‐initiated research grants from PureTech and Regeneron corporations. Dr. Dayan serves as a paid consultant for Stryker Corporation and a director of Welwaze Medical LLC without receiving any payment but minor shareholder.

## Supporting information

Figure S1. (A) mRNA expression changes in the normal and lymphedematous limb (*labelled LE)* of patients with unilateral BCRL. Each circle represents an individual patient (*N* = 12–14). (B) Correlations between TGF‐β1 mRNA expression and patient/disease factors. Abbreviations: BCRL, breast cancer‐related lymphedema; TGF‐β1, transforming growth factor‐beta 1
Figure S2. mRNA expression changes in the normal and lymphedematous limbs (*labelled LE)* of patients with unilateral BCRL. Each circle represents an individual patient (*N* = 12–14). Abbreviation: BCRL, breast cancer‐related lymphedema
Figure S3. (A) mRNA expression of TGF‐β downstream signaling mediators. Relative change to isotype control‐treated mice is shown. Each circle represents an individual animal (*N* = 5). Genes shown in the green‐ and red‐shaded zones represent upregulated (*green*) and downregulated (*red*) molecules. *p < .05, **p < .01. (B) mRNA expression of lymphatic genes and lymphangiogenic growth factors. Relative change to isotype control‐treated mice is shown. Genes shown in the red‐shaded zone represent downregulated molecules. Each circle represents an individual animal (*N* = 5). Genes shown in the green‐ and red‐shaded zones represent upregulated (*green*) and downregulated (*red*) molecules. *p < .05, **p < .01. (C) mRNA expression of TGF‐ β1 by flow‐sorted CD11b+ cells, CD4+ cells and stromal cells harvested from mice with tail lymphedema treated with isotype control antibodies. Each circle represents an average of two separate qPCR experiments per animal (*N* = 3 animals). (D) Representative flow cytometry and gating plan for Figure 3E. (E) Representative flow cytometry and gating plan for Figure 3F. Abbreviations: TGF‐β1, transforming growth factor‐beta 1; qPCR, quantitative polymerase chain reaction
Figure S4. (A) Quantification of TGF‐β1 and VEGF‐C in control and LE lysate using ELISA. Each circle represents an average of two ELISAs per animal (*N* = 5). (B) Proliferation of NIH3T3 fibroblasts 72 h after stimulation with normal media, LE lysate, LE lysate with a p38 inhibitor and LE lysate with a Smad inhibitor. Each circle represents an individual experiment for each condition. Abbreviations: TGF‐β1, transforming growth factor‐beta 1; VEGF‐C, vascular endothelial growth factor‐C; LE, lymphedema; ELISA, enzyme‐linked immunosorbent assay
Figure S5. (A) *Left panel*: Representative IF localisation of pSmad3 (*red*) and LYVE‐1 (*green*) in lymph node LECs of WT and LEC^DN‐TBRII^ mice. Yellow arrows point towards cell nuclei showing the presence or absence of pSmad3. *Right panel*: Quantification of the number of LYVE‐1+/SMAD3+ cells. Each circle represents an average of counts from three HPF views per animal (*N* = 4). (B) Representative IF localisation of CD31 (*red*) and LYVE‐1 (*green*) in corneal sections of WT and LEC^DN‐TBRII^ mice after suture placement. (C) Quantification of branch points in corneal sections of WT and LEC^DN‐TBRII^ mice. Each circle represents an average of three HPF views per animal (*N* = 5). (D) Quantification of TGF‐β1 protein in WT and LEC^DN‐TBRII^ mouse tails. Each circle represents an average of duplicate ELISAs per animal (*N* = 5). (E) Representative IF localisation of LYVE‐1 (*green*) showing bridging lymphatic vessels in LEC^DN‐TBRII^ but not WT mice. (F) Co‐expression of lymphatic markers LYVE‐1 (*green*), VEGFR‐3 (*gra*y) and podoplanin (*red*) on lymphatic vessels in a representative section of the mouse tail. Abbreviations: IF, immunofluorescence; LECs, lymphatic endothelial cells; WT, wild type; HPF, high‐power field; TGF‐β1, transforming growth factor‐beta 1; ELISA, enzyme‐linked immunosorbent assay; VEGFR‐3, vascular endothelial growth factor receptor‐3
Figure S6. (A) Representative image of a vehicle and PFD‐treated mouse tail. (B) Representative IF localisation of type I collagen (Col1; *red*) and LYVE‐1 (*green*) in vehicle and PFD‐treated mouse tails. (C) *Left panel*: Representative IF localisation of TGF‐β1 (*red*) in vehicle and PFD‐treated mouse tails. *Right panel*: Quantification of TGF‐β1 area in IF stained sections. Each circle represents an average of three HPF views from 7 animals. (D) Representative IF localisation of pSmad3 (*red*) and LYVE‐1 (*green*) in vehicle and PFD‐treated mouse tails. (E) mRNA expression of TGF‐β downstream signaling genes. Relative change to vehicle control‐treated mice is shown. Each circle represents an individual animal (*N* = 6). Genes shown in the green‐ and red‐shaded zones represent upregulated (*green*) and downregulated (*red*) molecules. *p < .05, **p < .01. (F) Protein expression of IFN‐g, IL13, VEGF‐C and VEGF‐A in vehicle and PFD‐treated mouse tails using ELISA. Each circle represents an average of duplicate ELISAs per animal (*N* = 4–7). (G) *Left panel*: Representative IF localisation of F4/80 (*red*) and LYVE‐1 (*green*) in vehicle and PFD‐treated mouse tails. *Right panel*: Quantification of perilymphatic CDF4/80^+^ cells. Each circle represents an average of cell counts in three HPF views from 9 animals. Abbreviations: PFD, pirfenidone; IF, immunofluorescence; TGF‐β1, transforming growth factor‐beta 1; HPF, high‐power field; IFN‐g, interferon‐gamma; IL13, interleukin 13; VEGF‐C, ‐A, vascular endothelial growth factor‐C, ‐A; ELISA, enzyme‐linked immunosorbent assay
Figure S7. (A) *Left panel*: Representative IF localisation of iNOS (*red*) and LYVE‐1 (*green*) in vehicle and PFD‐treated mouse tails. *Right panel*: Quantification of iNOS^+^ cells in IF stained sections. Each circle represents an average of three HPF views per animal (*N* = 8). (B) Experimental plan for animals treated with PLND and treated with PFD ± TGF‐β1 mAb. (C) Light intensity plots were obtained using ICG lymphangiography in animals that had undergone PLND and were treated with PFD ± TGF‐β1 mAb. (D) Quantification of dermal backflow in animals that had undergone PLND and were treated with PFD ± TGF‐β1 mAb. Each circle represents an average of two measurements per animal (*N* = 5). (E) Quantification of packets/min (collecting lymphatic pumping) in animals that had undergone PLND and were treated with PFD ± TGF‐β1 mAb. Each circle represents an average of two measurements per animal (*N* = 5). Abbreviations: IF, immunofluorescence; HPF, high‐power field; PLND, popliteal lymph node dissections; PFD, pirfenidone; TGF‐β1, transforming growth factor‐beta 1; mAb, monoclonal antibody; ICG, indocyanine green
