# Supplementary material for: TGF‐β1 mediates pathologic changes of secondary lymphedema by promoting fibrosis and inflammation
Source: Clin Transl Med. 2022 Jun 2;12(6):e758. doi: 10.1002/ctm2.758 (PMC9160979; doi:10.1002/ctm2.758)
Supplement: Supplementary file 1 — Figure S1. (A) mRNA expression changes in the normal and lymphedematous limb (labelled LE) of patients with unilateral BCRL. Each circle represents an individual patient (N = 12–14). (B) Correlations between TGF‐β1 mRNA expression and patient/disease factors. Abbreviations: BCRL, breast cancer‐related lymphedema; TGF‐β1, transforming growth factor‐beta 1 Figure S2. mRNA expression changes in the normal and lymphedematous limbs (labelled LE) of patients with unilateral BCRL. Each circle represents an individual patient (N = 12–14). Abbreviation: BCRL, breast cancer‐related lymphedema Figure S3. (A) mRNA expression of TGF‐β downstream signaling mediators. Relative change to isotype control‐treated mice is shown. Each circle represents an individual animal (N = 5). Genes shown in the green‐ and red‐shaded zones represent upregulated (green) and downregulated (red) molecules. *p < .05, **p < .01. (B) mRNA expression of lymphatic genes and lymphangiogenic growth factors. Relative change to isotype control‐treated mice is shown. Genes shown in the red‐shaded zone represent downregulated molecules. Each circle represents an individual animal (N = 5). Genes shown in the green‐ and red‐shaded zones represent upregulated (green) and downregulated (red) molecules. *p < .05, **p < .01. (C) mRNA expression of TGF‐ β1 by flow‐sorted CD11b+ cells, CD4+ cells and stromal cells harvested from mice with tail lymphedema treated with isotype control antibodies. Each circle represents an average of two separate qPCR experiments per animal (N = 3 animals). (D) Representative flow cytometry and gating plan for Figure 3E. (E) Representative flow cytometry and gating plan for Figure 3F. Abbreviations: TGF‐β1, transforming growth factor‐beta 1; qPCR, quantitative polymerase chain reaction Figure S4. (A) Quantification of TGF‐β1 and VEGF‐C in control and LE lysate using ELISA. Each circle represents an average of two ELISAs per animal (N = 5). (B) Proliferation of NIH3T3 fibroblasts 72 h afte [file CTM2-12-e758-s001.pptx]

## Slide 1
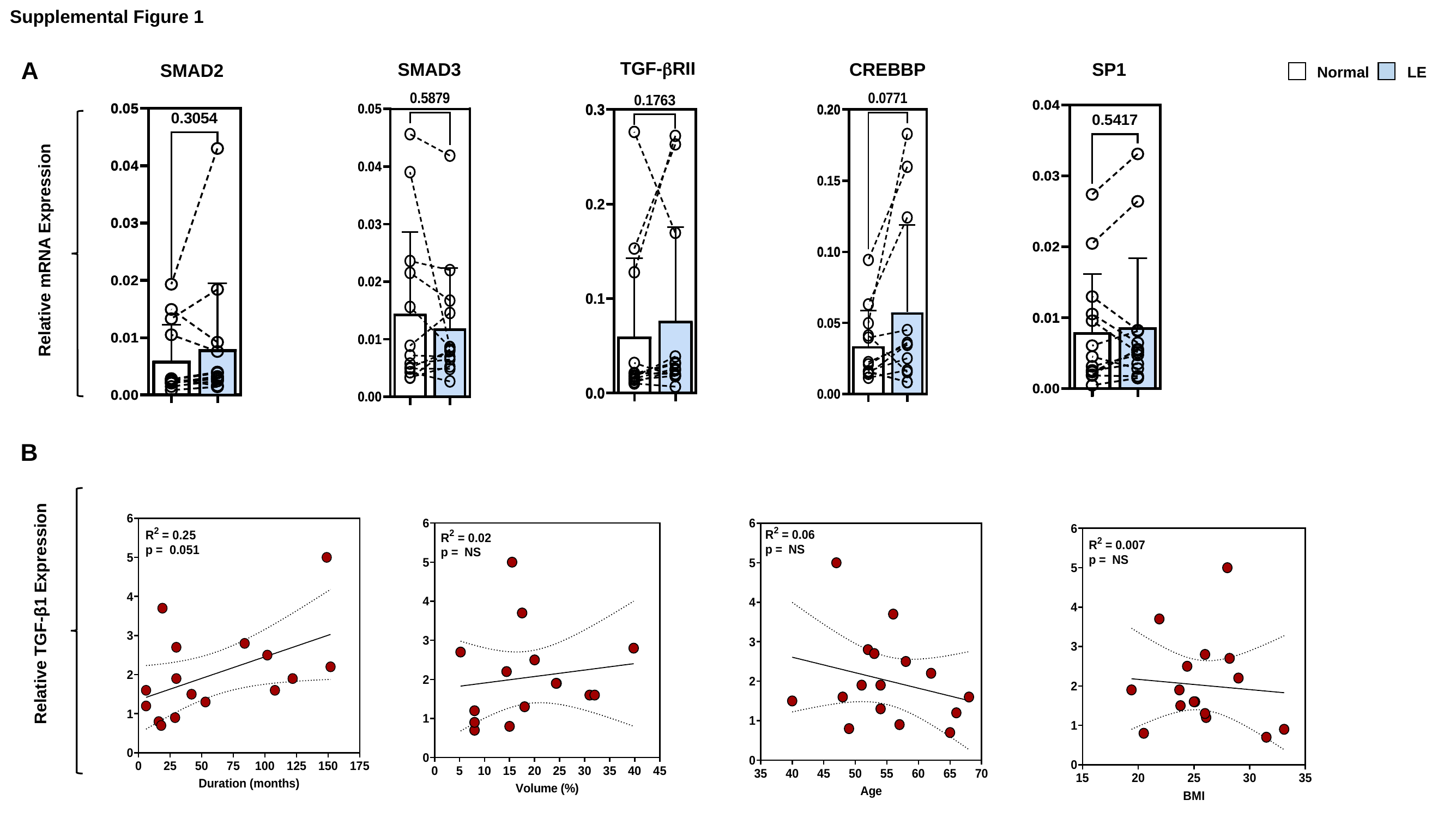

Supplemental Figure 1
TGF-bRII
SP1
SMAD3
CREBBP
SMAD2
A
Normal
LE
Relative mRNA Expression
B
Relative TGF-β1 Expression

## Slide 2
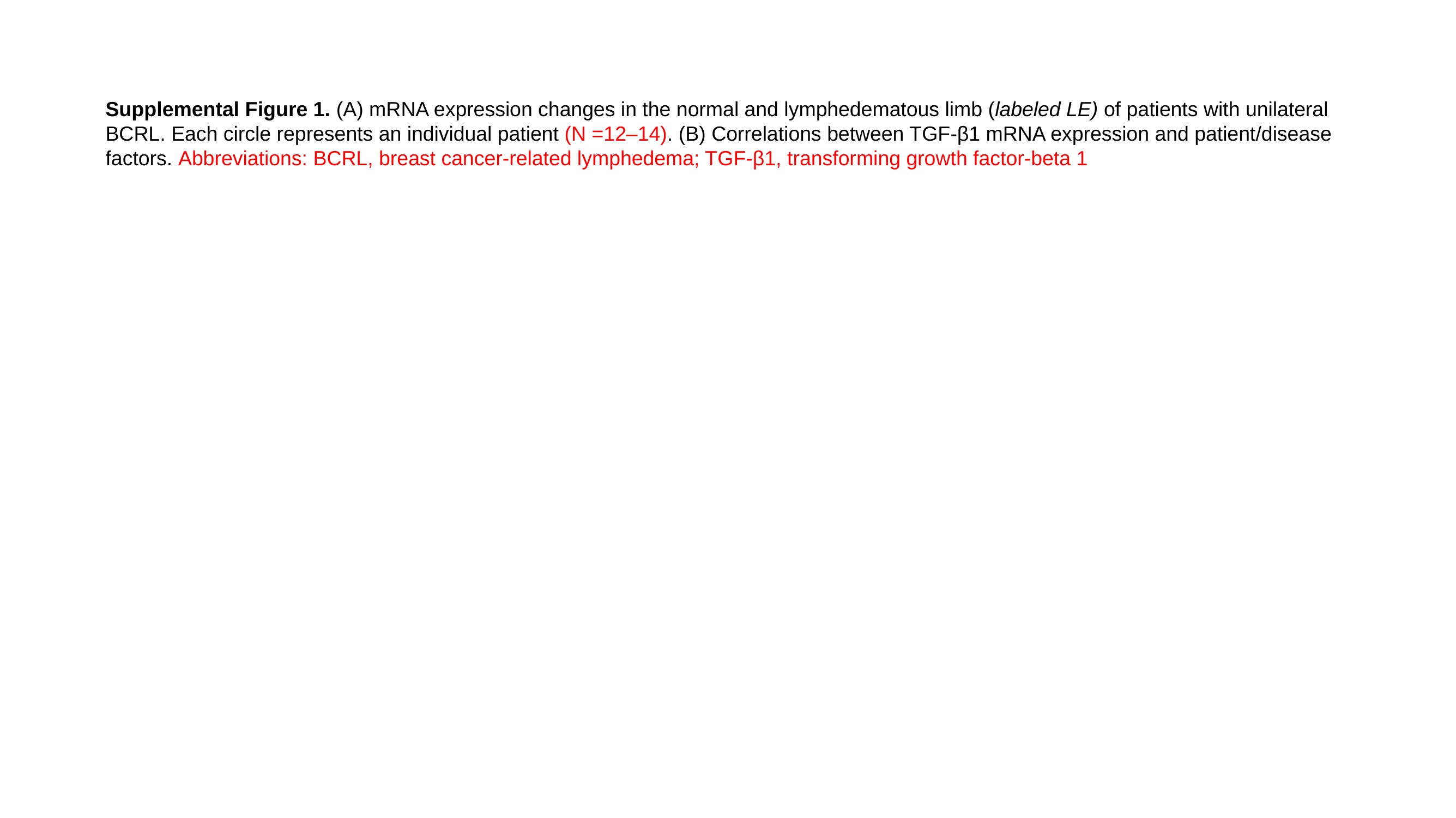

Supplemental Figure 1. (A) mRNA expression changes in the normal and lymphedematous limb (labeled LE) of patients with unilateral BCRL. Each circle represents an individual patient (N =12–14). (B) Correlations between TGF-β1 mRNA expression and patient/disease factors. Abbreviations: BCRL, breast cancer-related lymphedema; TGF-β1, transforming growth factor-beta 1

## Slide 3
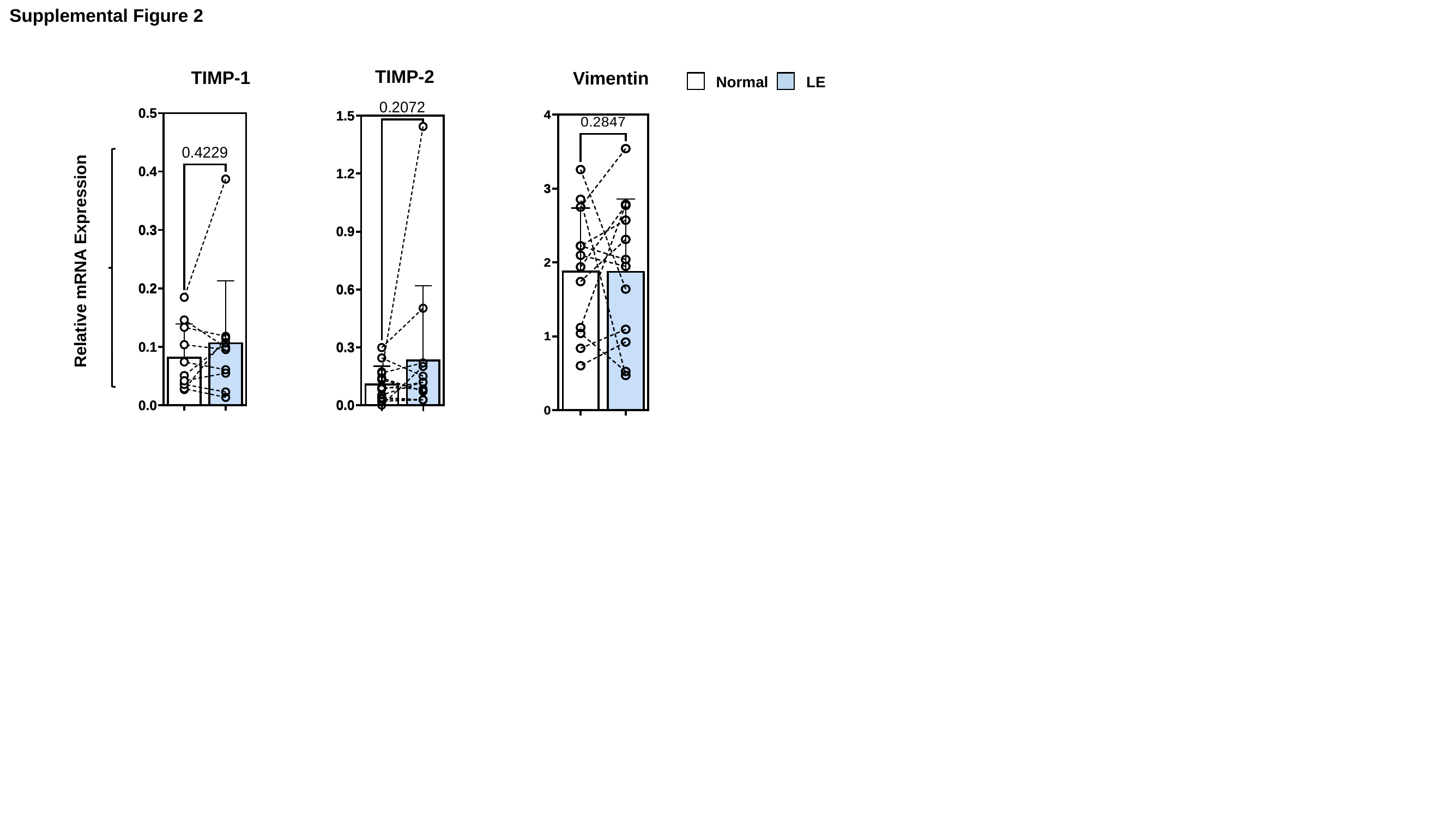

Supplemental Figure 2
TIMP-2
TIMP-1
Vimentin
Normal
LE
Relative mRNA Expression

## Slide 4
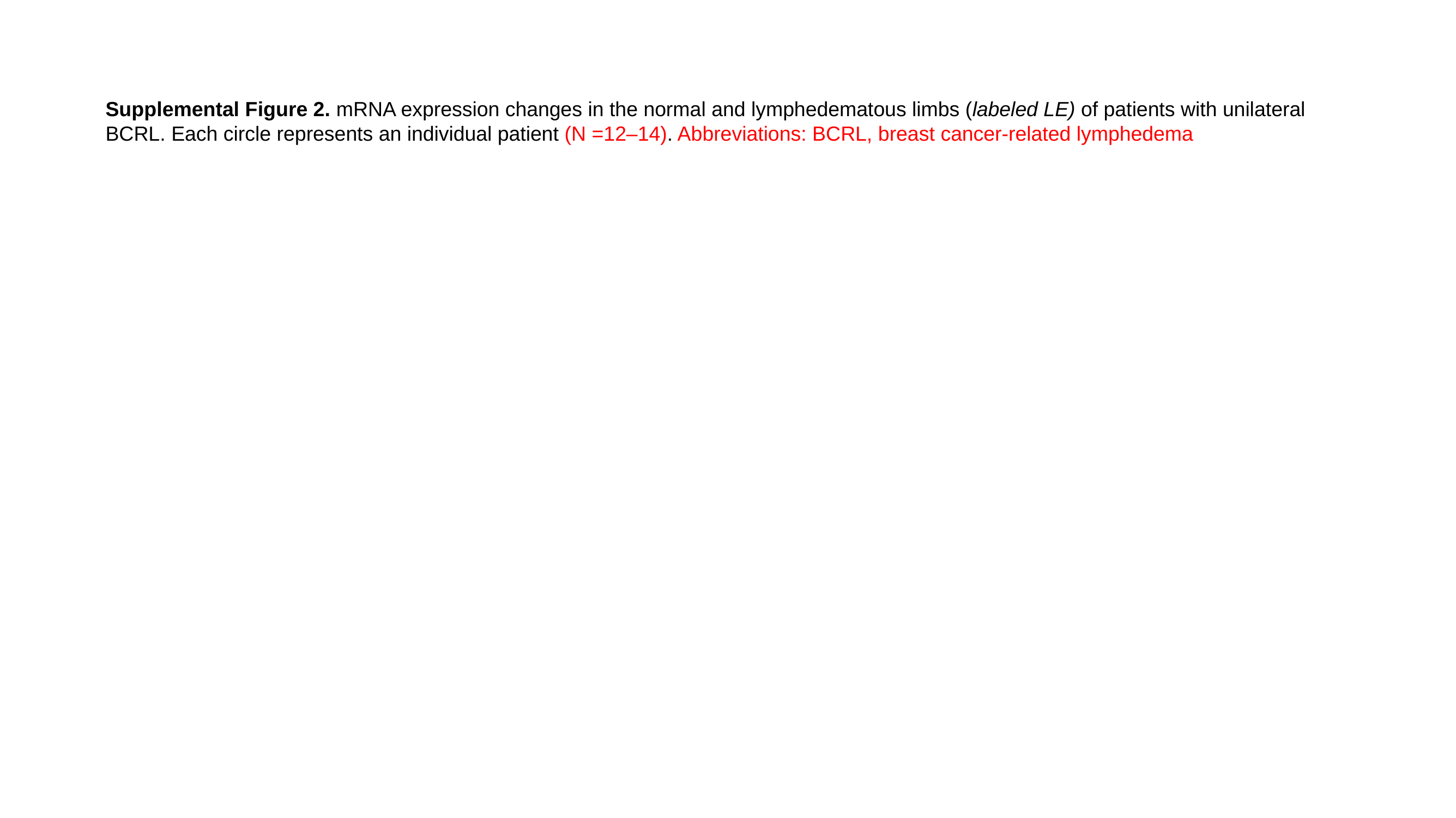

Supplemental Figure 2. mRNA expression changes in the normal and lymphedematous limbs (labeled LE) of patients with unilateral BCRL. Each circle represents an individual patient (N =12–14). Abbreviations: BCRL, breast cancer-related lymphedema

## Slide 5
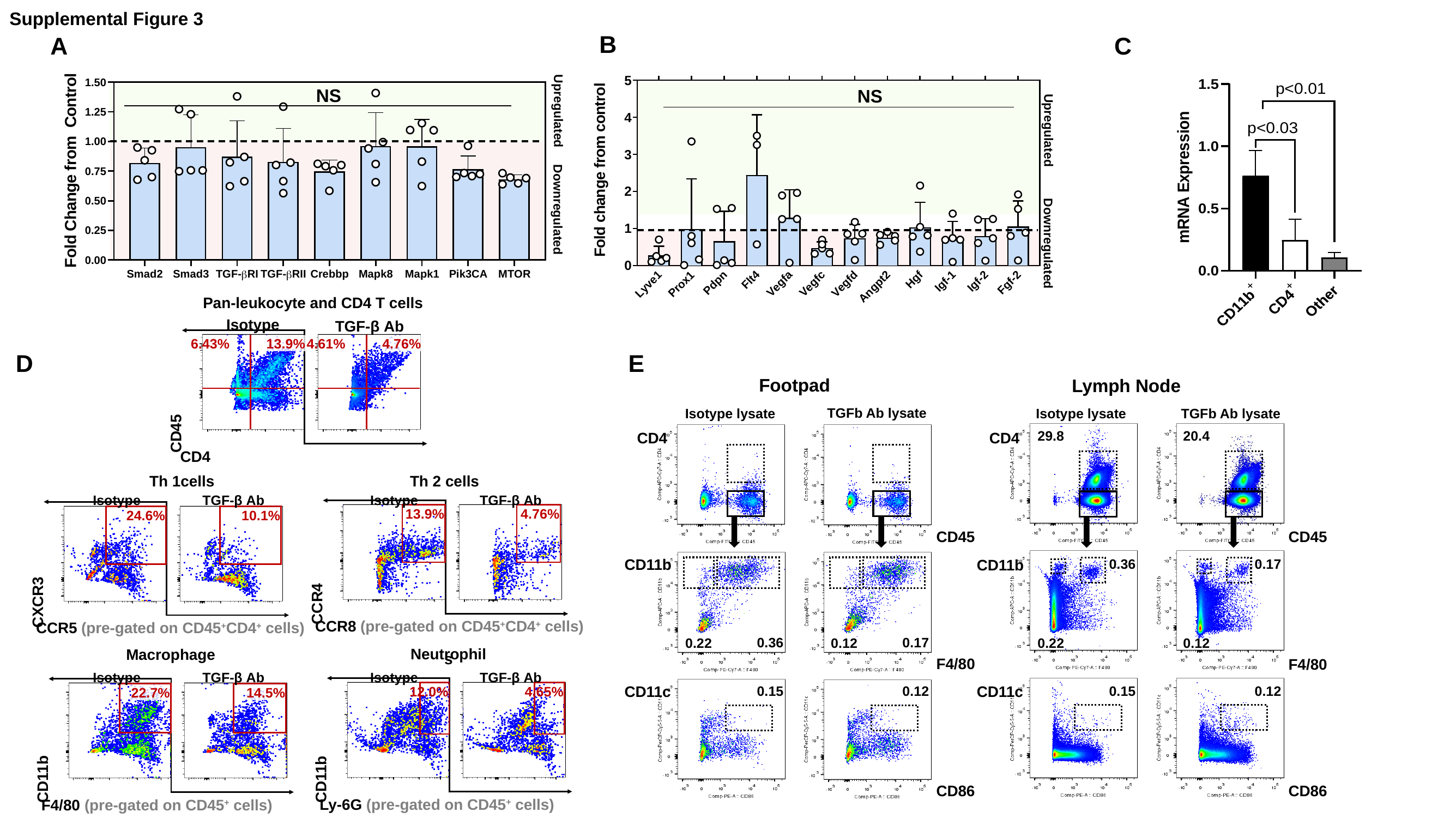

Supplemental Figure 3
B
A
C
NS
NS
Upregulated
Upregulated
Downregulated
Downregulated
Pan-leukocyte and CD4 T cells
Isotype
TGF-β Ab
6.43%
13.9%
4.61%
4.76%
CD45
CD4
Th 1cells
Th 2 cells
13.9%
4.76%
CCR4
CCR8 (pre-gated on CD45+CD4+ cells)
Isotype
TGF-β Ab
Isotype
TGF-β Ab
24.6%
10.1%
CXCR3
CCR5 (pre-gated on CD45+CD4+ cells)
Macrophage
Neutrophils
12.0%
4.65%
CD11b
Ly-6G (pre-gated on CD45+ cells)
22.7%
14.5%
CD11b
F4/80 (pre-gated on CD45+ cells)
Isotype
TGF-β Ab
Isotype
TGF-β Ab
D
E
Footpad
TGFb Ab lysate
Isotype lysate
CD4
CD45
CD11b
0.36
0.17
0.22
0.12
F4/80
CD11c
0.15
0.12
CD86
Lymph Node
TGFb Ab lysate
Isotype lysate
29.8
20.4
CD4
CD45
CD11b
0.36
0.17
0.22
0.12
F4/80
CD11c
0.15
0.12
CD86

## Slide 6
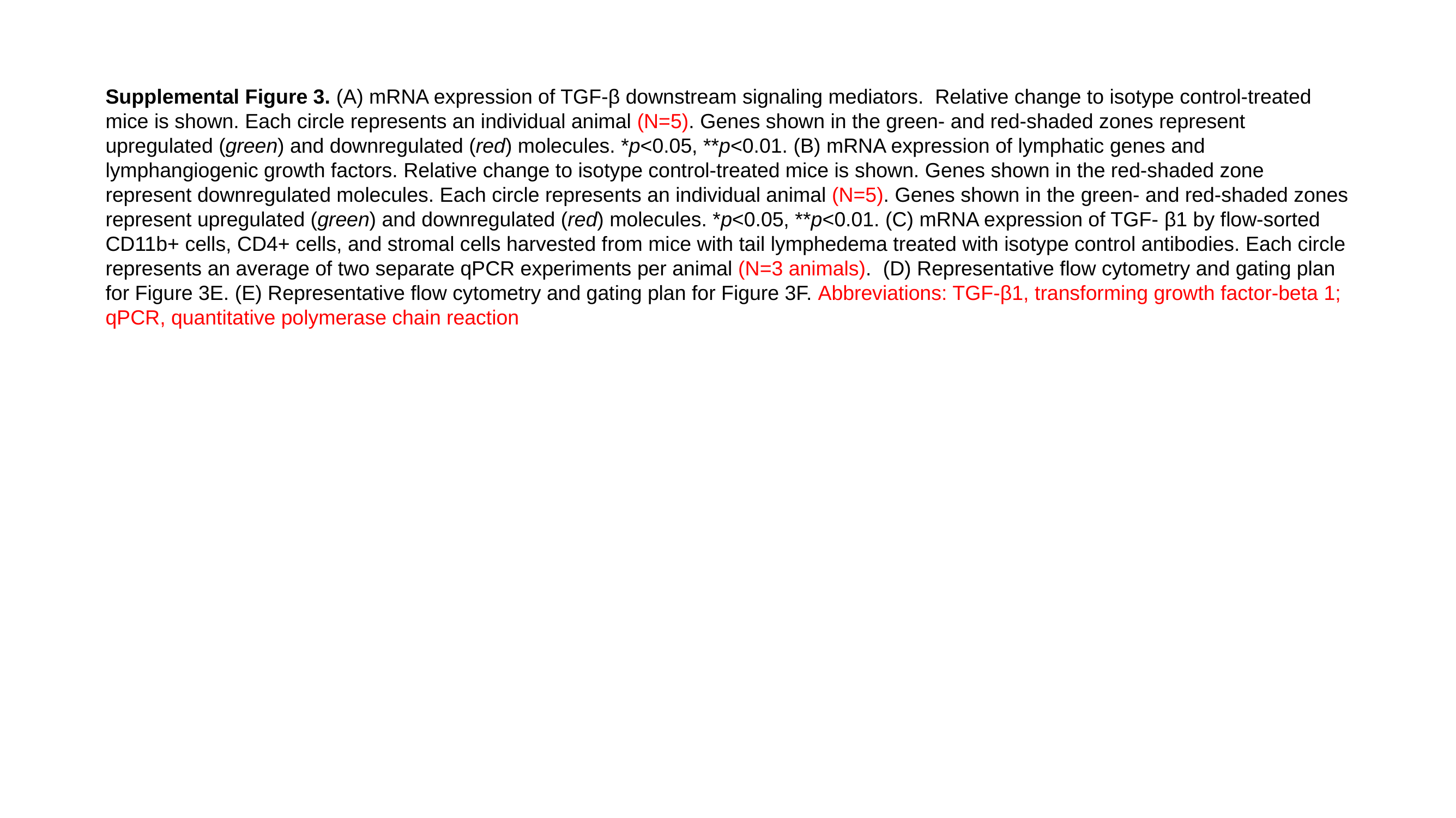

Supplemental Figure 3. (A) mRNA expression of TGF-β downstream signaling mediators. Relative change to isotype control-treated mice is shown. Each circle represents an individual animal (N=5). Genes shown in the green- and red-shaded zones represent upregulated (green) and downregulated (red) molecules. *p<0.05, **p<0.01. (B) mRNA expression of lymphatic genes and lymphangiogenic growth factors. Relative change to isotype control-treated mice is shown. Genes shown in the red-shaded zone represent downregulated molecules. Each circle represents an individual animal (N=5). Genes shown in the green- and red-shaded zones represent upregulated (green) and downregulated (red) molecules. *p<0.05, **p<0.01. (C) mRNA expression of TGF- β1 by flow-sorted CD11b+ cells, CD4+ cells, and stromal cells harvested from mice with tail lymphedema treated with isotype control antibodies. Each circle represents an average of two separate qPCR experiments per animal (N=3 animals). (D) Representative flow cytometry and gating plan for Figure 3E. (E) Representative flow cytometry and gating plan for Figure 3F. Abbreviations: TGF-β1, transforming growth factor-beta 1; qPCR, quantitative polymerase chain reaction

## Slide 7
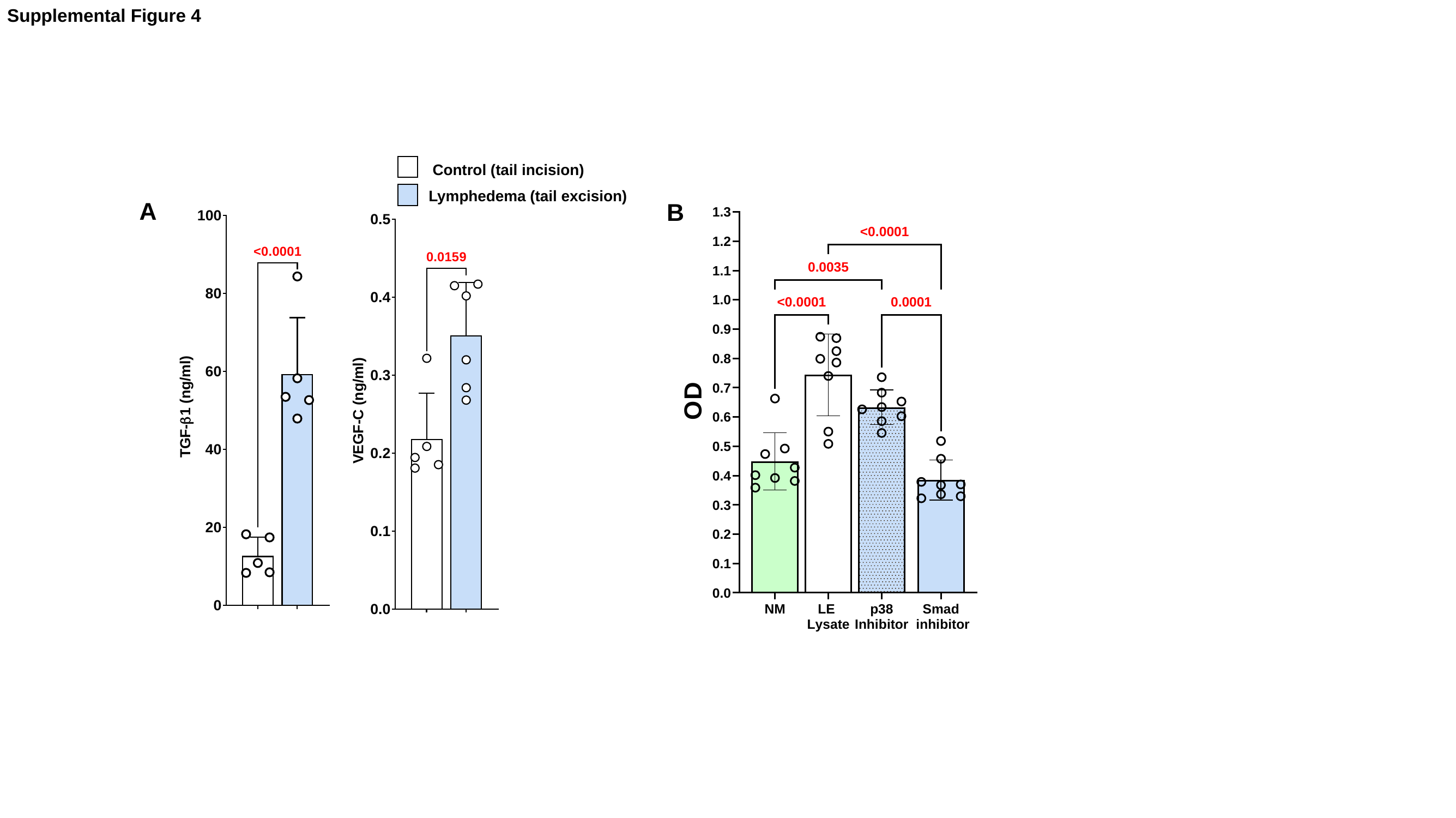

Supplemental Figure 4
Control (tail incision)
Lymphedema (tail excision)
A
B

## Slide 8
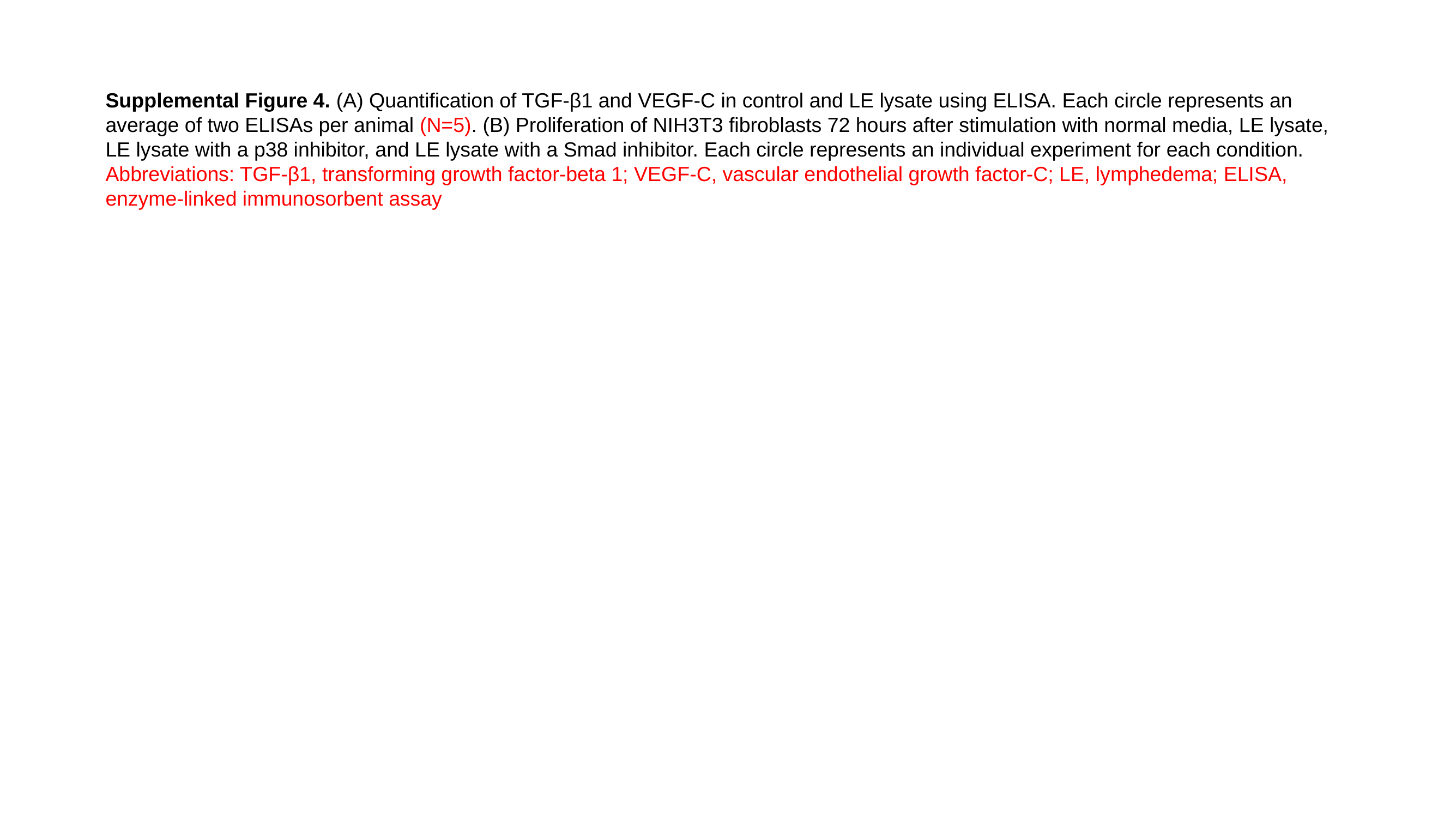

Supplemental Figure 4. (A) Quantification of TGF-β1 and VEGF-C in control and LE lysate using ELISA. Each circle represents an average of two ELISAs per animal (N=5). (B) Proliferation of NIH3T3 fibroblasts 72 hours after stimulation with normal media, LE lysate, LE lysate with a p38 inhibitor, and LE lysate with a Smad inhibitor. Each circle represents an individual experiment for each condition. Abbreviations: TGF-β1, transforming growth factor-beta 1; VEGF-C, vascular endothelial growth factor-C; LE, lymphedema; ELISA, enzyme-linked immunosorbent assay

## Slide 9
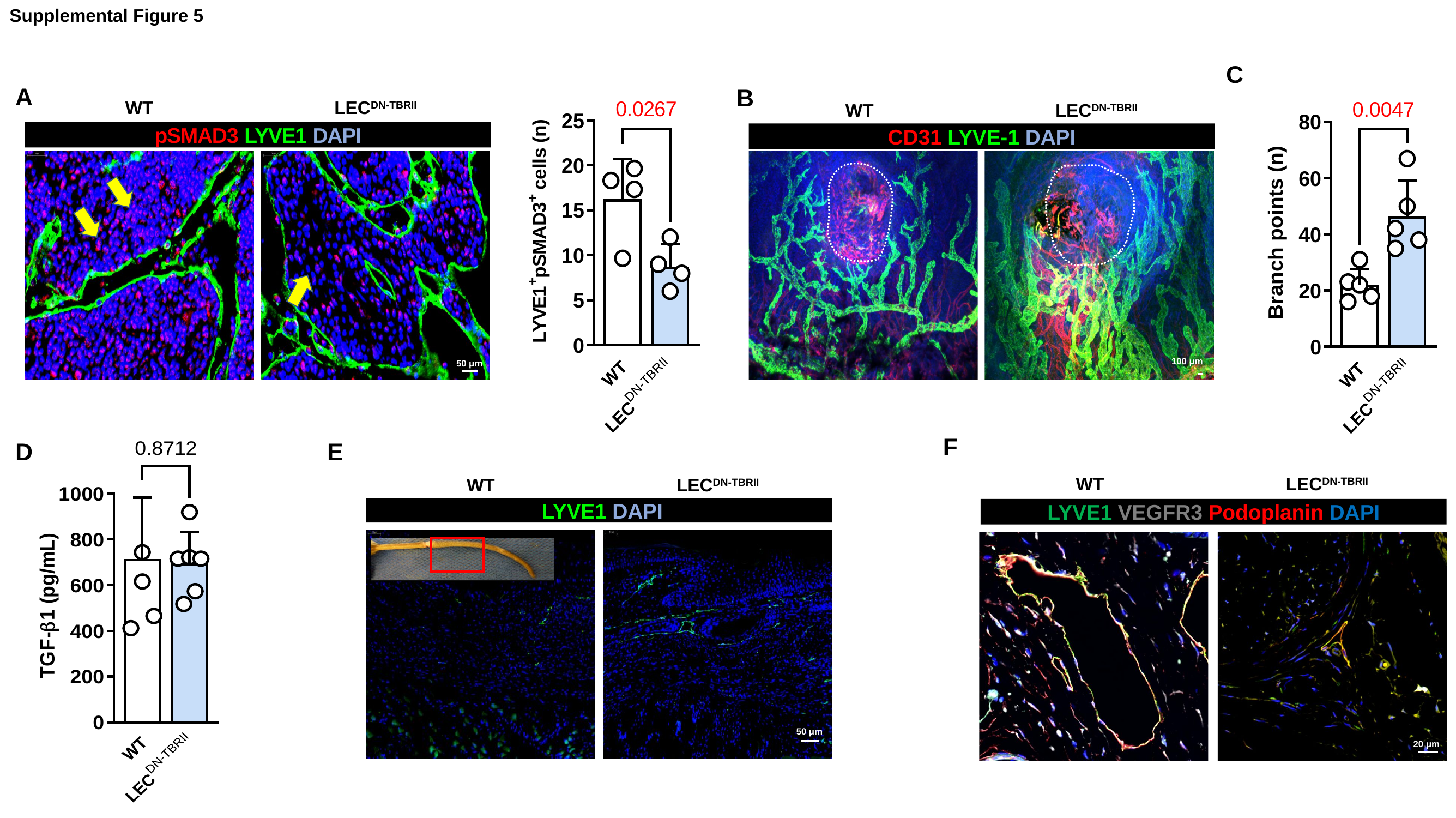

Supplemental Figure 5
C
A
B
WT
LECDN-TBRII
WT
LECDN-TBRII
pSMAD3 LYVE1 DAPI
CD31 LYVE-1 DAPI
100 μm
50 μm
F
E
D
WT
LECDN-TBRII
LYVE1 VEGFR3 Podoplanin DAPI
20 μm
WT
LECDN-TBRII
 LYVE1 DAPI
50 μm

## Slide 10
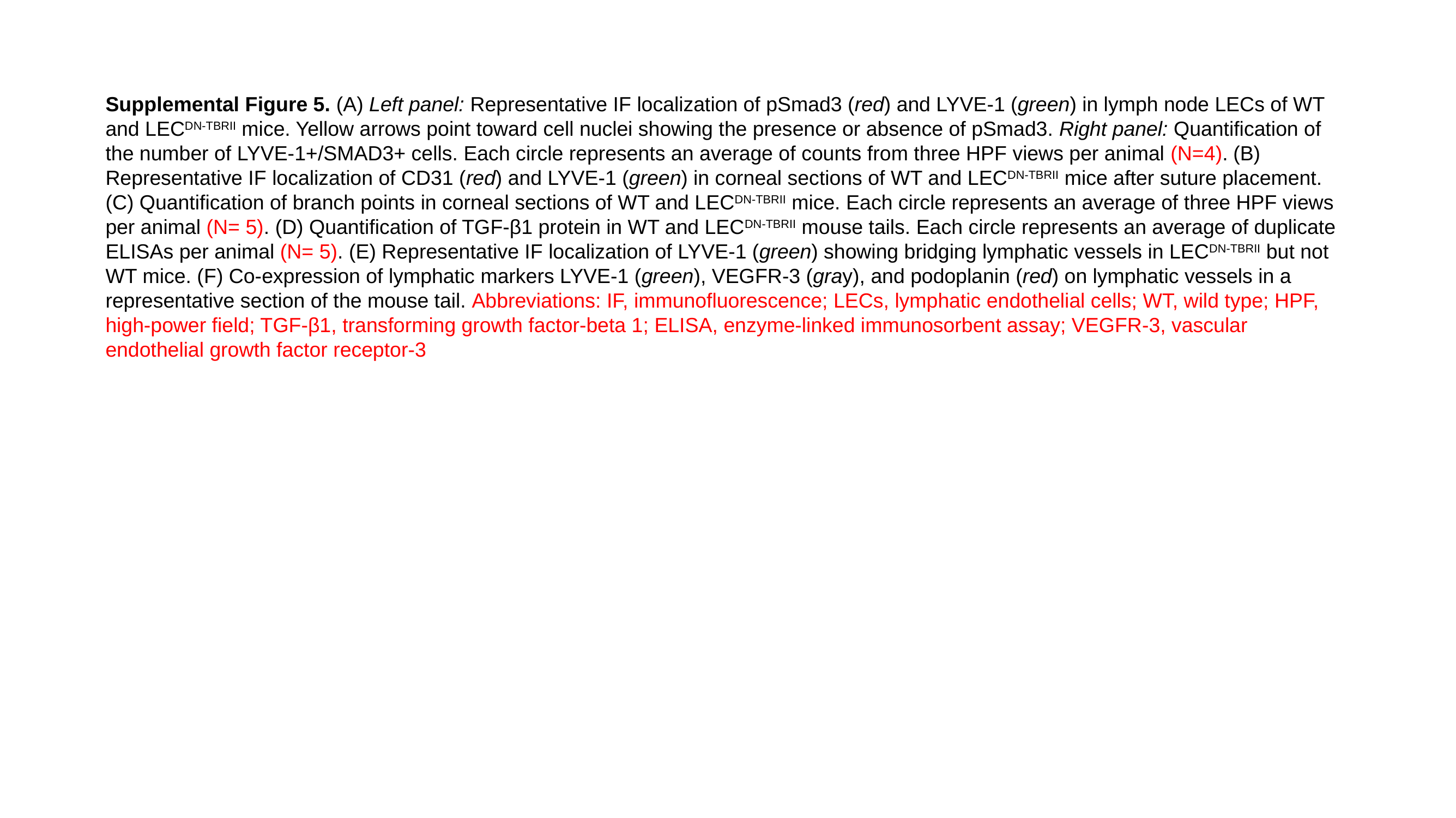

Supplemental Figure 5. (A) Left panel: Representative IF localization of pSmad3 (red) and LYVE-1 (green) in lymph node LECs of WT and LECDN-TBRII mice. Yellow arrows point toward cell nuclei showing the presence or absence of pSmad3. Right panel: Quantification of the number of LYVE-1+/SMAD3+ cells. Each circle represents an average of counts from three HPF views per animal (N=4). (B) Representative IF localization of CD31 (red) and LYVE-1 (green) in corneal sections of WT and LECDN-TBRII mice after suture placement. (C) Quantification of branch points in corneal sections of WT and LECDN-TBRII mice. Each circle represents an average of three HPF views per animal (N= 5). (D) Quantification of TGF-β1 protein in WT and LECDN-TBRII mouse tails. Each circle represents an average of duplicate ELISAs per animal (N= 5). (E) Representative IF localization of LYVE-1 (green) showing bridging lymphatic vessels in LECDN-TBRII but not WT mice. (F) Co-expression of lymphatic markers LYVE-1 (green), VEGFR-3 (gray), and podoplanin (red) on lymphatic vessels in a representative section of the mouse tail. Abbreviations: IF, immunofluorescence; LECs, lymphatic endothelial cells; WT, wild type; HPF, high-power field; TGF-β1, transforming growth factor-beta 1; ELISA, enzyme-linked immunosorbent assay; VEGFR-3, vascular endothelial growth factor receptor-3

## Slide 11
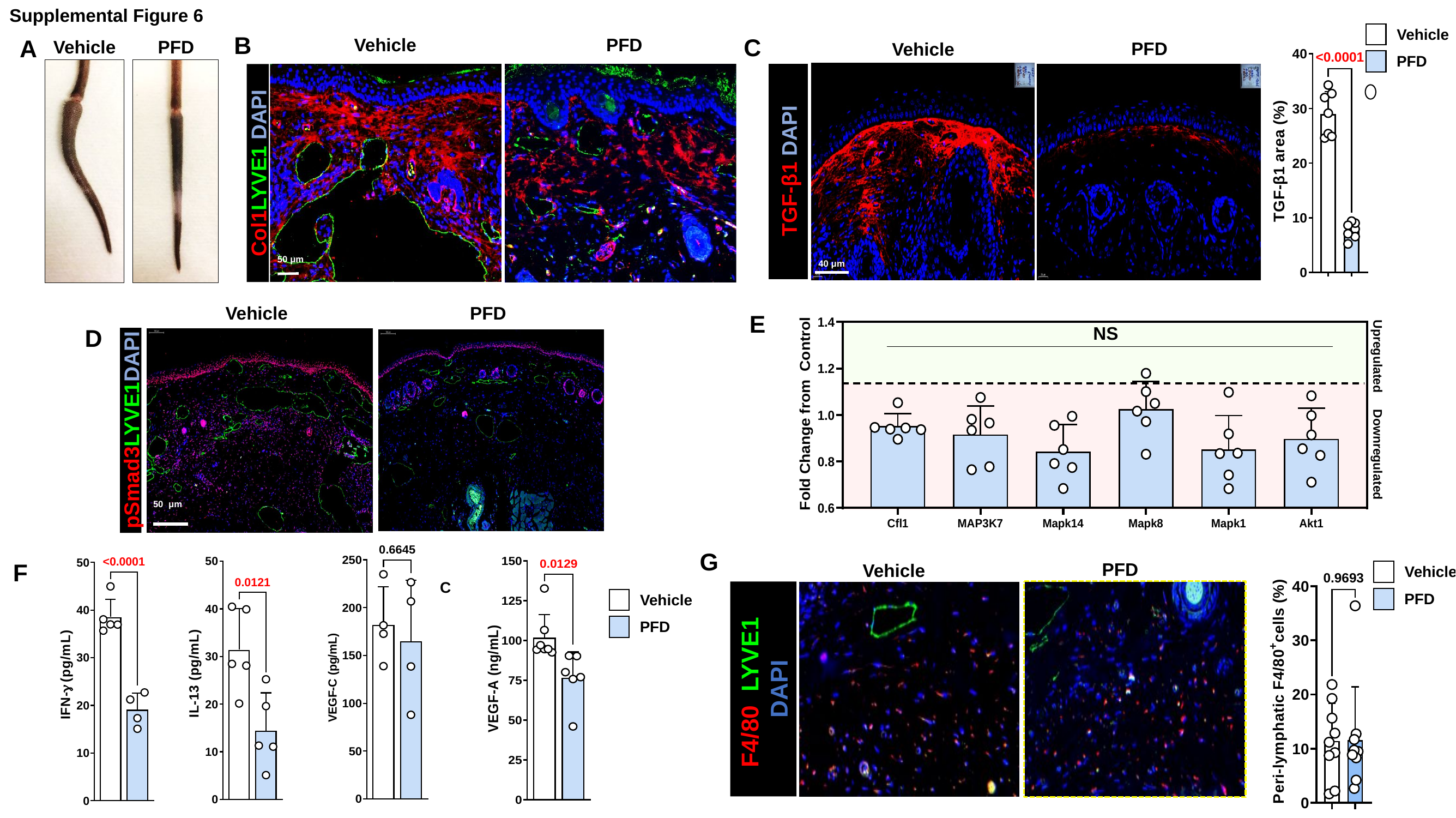

Supplemental Figure 6
Vehicle
PFD
B
C
A
Vehicle
PFD
50 μm
Col1LYVE1 DAPI
Vehicle
PFD
PFD
Vehicle
TGF-β1 DAPI
40 μm
NS
Vehicle
PFD
50 μm
pSmad3LYVE1DAPI
E
D
Upregulated
Downregulated
G
F
PFD
Vehicle
PFD
Vehicle
C
Vehicle
PFD
F4/80 LYVE1 DAPI

## Slide 12
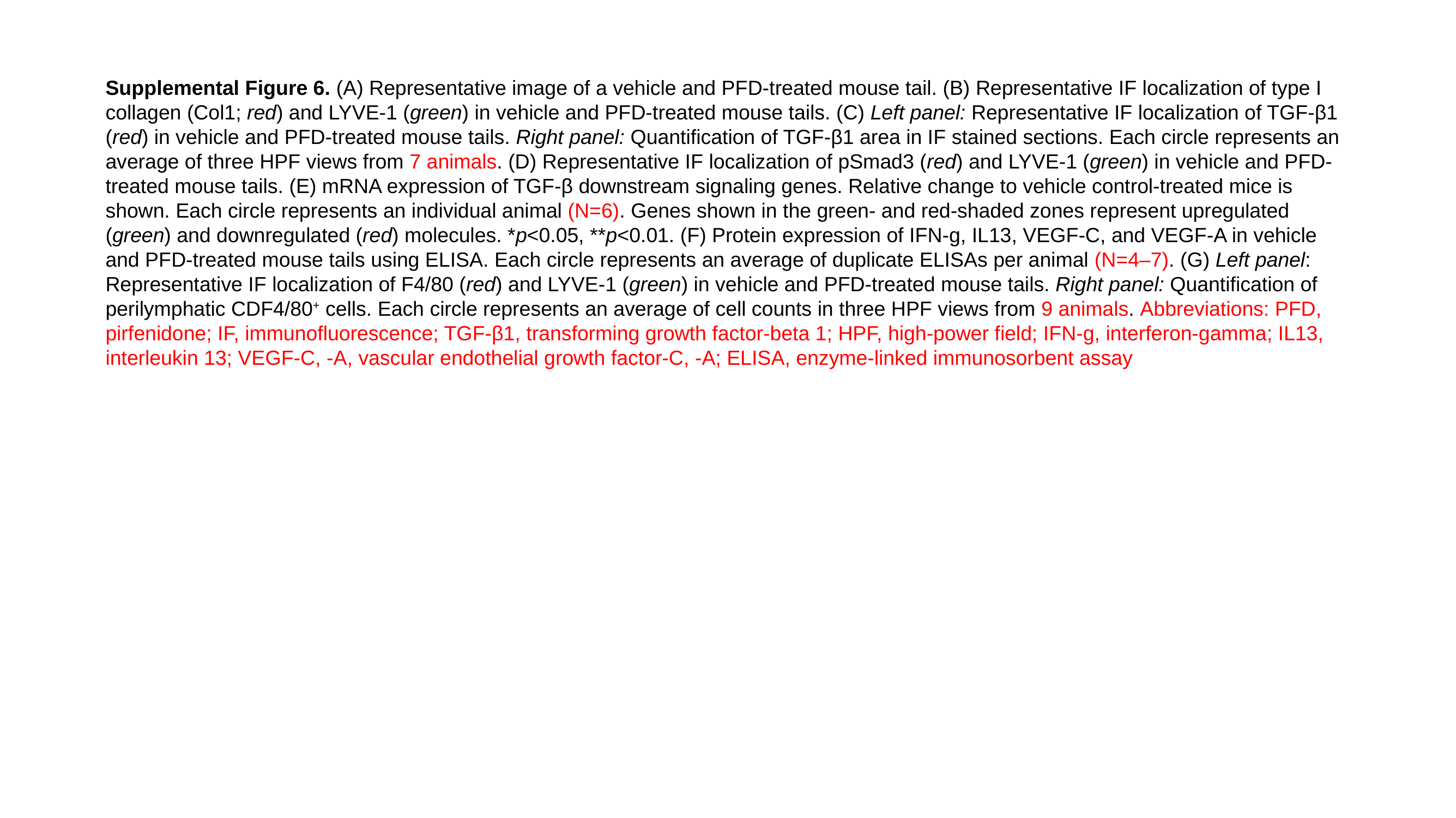

Supplemental Figure 6. (A) Representative image of a vehicle and PFD-treated mouse tail. (B) Representative IF localization of type I collagen (Col1; red) and LYVE-1 (green) in vehicle and PFD-treated mouse tails. (C) Left panel: Representative IF localization of TGF-β1 (red) in vehicle and PFD-treated mouse tails. Right panel: Quantification of TGF-β1 area in IF stained sections. Each circle represents an average of three HPF views from 7 animals. (D) Representative IF localization of pSmad3 (red) and LYVE-1 (green) in vehicle and PFD-treated mouse tails. (E) mRNA expression of TGF-β downstream signaling genes. Relative change to vehicle control-treated mice is shown. Each circle represents an individual animal (N=6). Genes shown in the green- and red-shaded zones represent upregulated (green) and downregulated (red) molecules. *p<0.05, **p<0.01. (F) Protein expression of IFN-g, IL13, VEGF-C, and VEGF-A in vehicle and PFD-treated mouse tails using ELISA. Each circle represents an average of duplicate ELISAs per animal (N=4–7). (G) Left panel: Representative IF localization of F4/80 (red) and LYVE-1 (green) in vehicle and PFD-treated mouse tails. Right panel: Quantification of perilymphatic CDF4/80+ cells. Each circle represents an average of cell counts in three HPF views from 9 animals. Abbreviations: PFD, pirfenidone; IF, immunofluorescence; TGF-β1, transforming growth factor-beta 1; HPF, high-power field; IFN-g, interferon-gamma; IL13, interleukin 13; VEGF-C, -A, vascular endothelial growth factor-C, -A; ELISA, enzyme-linked immunosorbent assay

## Slide 13
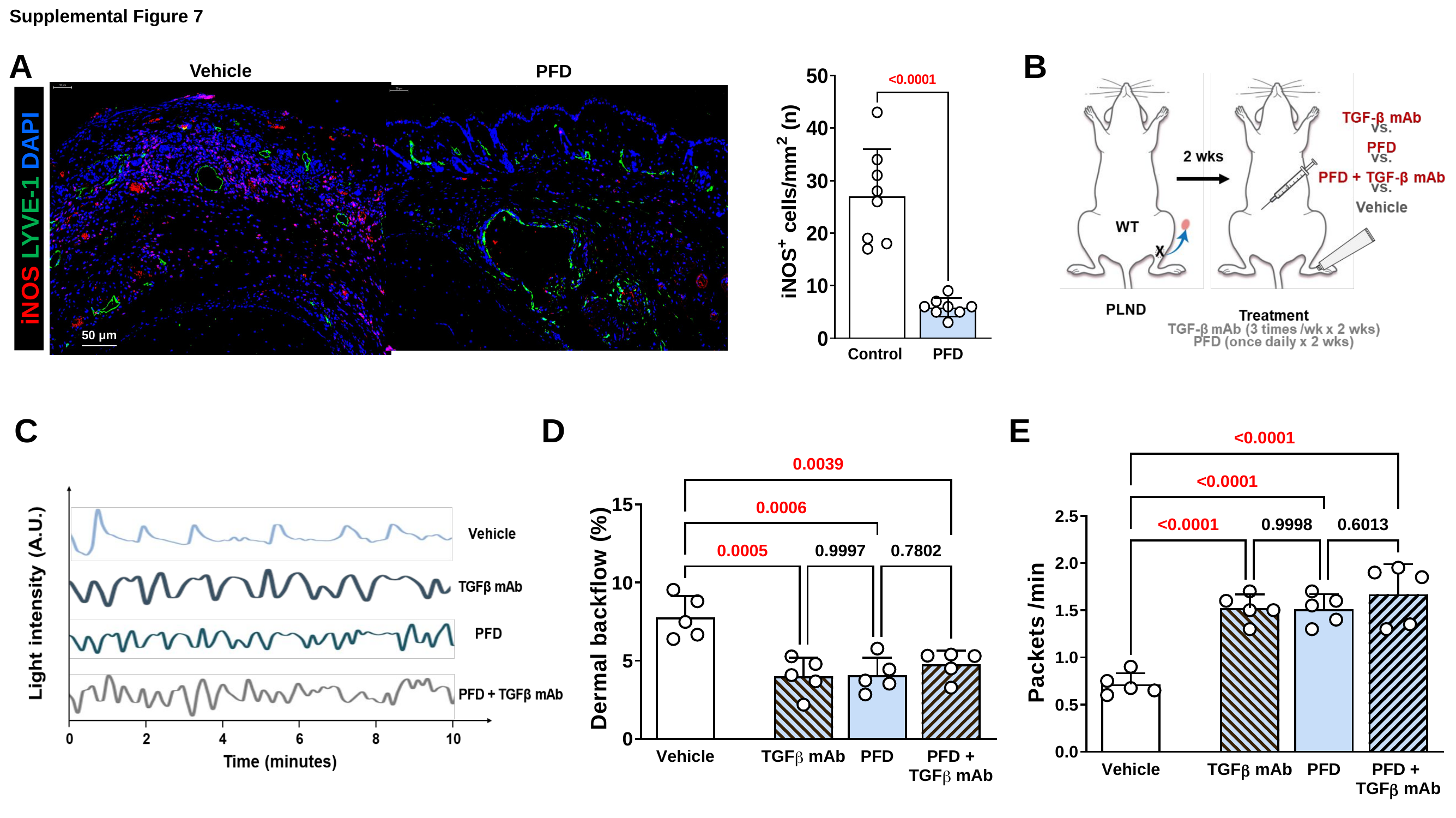

Supplemental Figure 7
A
B
Vehicle
PFD
iNOS LYVE-1 DAPI
50 μm
C
D
E

## Slide 14
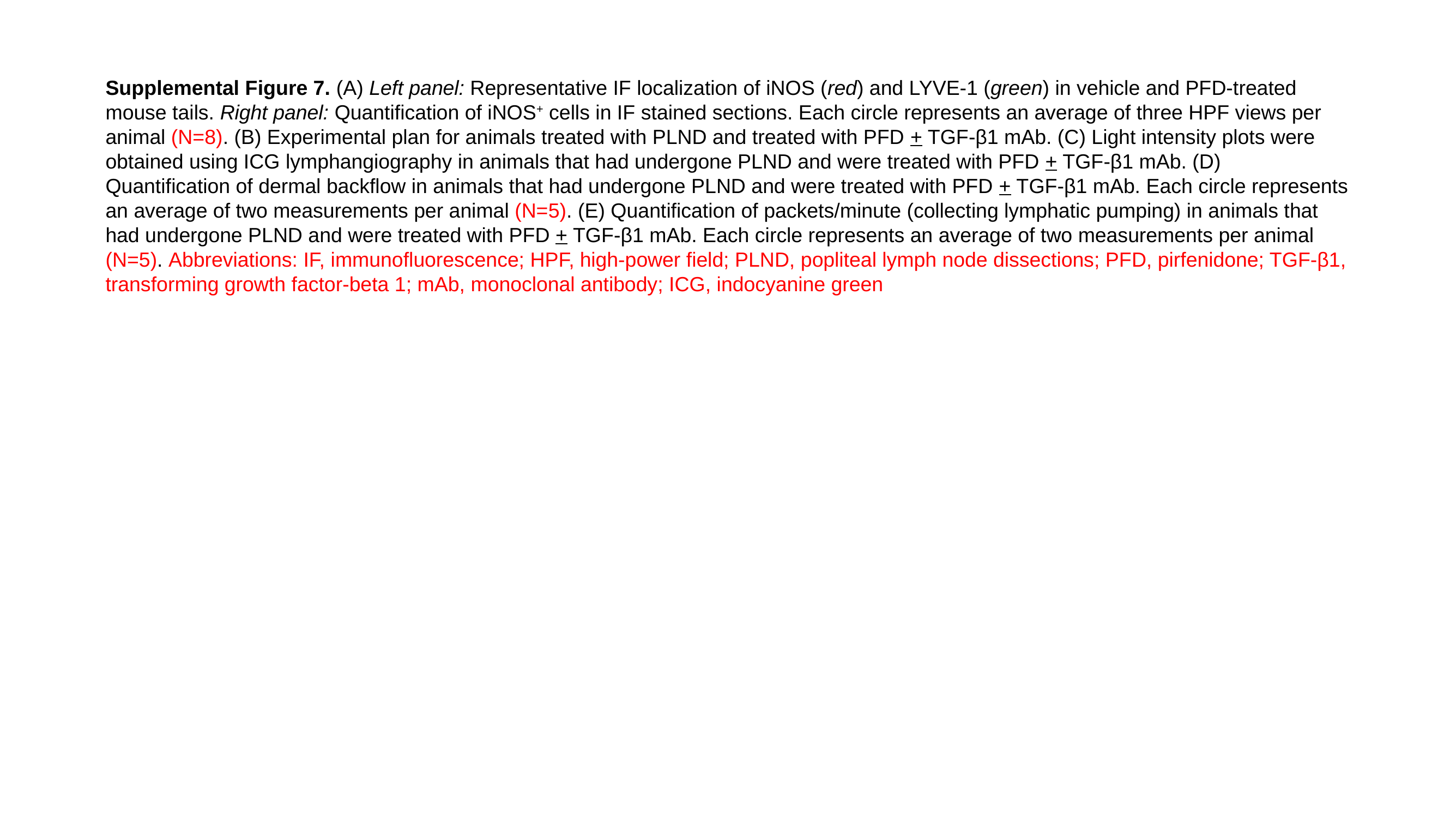

Supplemental Figure 7. (A) Left panel: Representative IF localization of iNOS (red) and LYVE-1 (green) in vehicle and PFD-treated mouse tails. Right panel: Quantification of iNOS+ cells in IF stained sections. Each circle represents an average of three HPF views per animal (N=8). (B) Experimental plan for animals treated with PLND and treated with PFD + TGF-β1 mAb. (C) Light intensity plots were obtained using ICG lymphangiography in animals that had undergone PLND and were treated with PFD + TGF-β1 mAb. (D) Quantification of dermal backflow in animals that had undergone PLND and were treated with PFD + TGF-β1 mAb. Each circle represents an average of two measurements per animal (N=5). (E) Quantification of packets/minute (collecting lymphatic pumping) in animals that had undergone PLND and were treated with PFD + TGF-β1 mAb. Each circle represents an average of two measurements per animal (N=5). Abbreviations: IF, immunofluorescence; HPF, high-power field; PLND, popliteal lymph node dissections; PFD, pirfenidone; TGF-β1, transforming growth factor-beta 1; mAb, monoclonal antibody; ICG, indocyanine green
